# Global Patterns of QALY and DALY Use in Surgical Cost-Utility Analyses: A Systematic Review

**DOI:** 10.1371/journal.pone.0148304

**Published:** 2016-02-10

**Authors:** Arturo J. Rios-Diaz, Jimmy Lam, Margarita S. Ramos, Andrea V. Moscoso, Patrick Vaughn, Cheryl K. Zogg, Edward J. Caterson

**Affiliations:** 1 Center for Surgery and Public Health, Harvard Medical School & Harvard School of Public Health, Department of Surgery, Brigham and Women’s Hospital, Boston, Massachusetts, United States of America; 2 Boston University, School of Medicine, Boston, Massachusetts, United States of America; 3 Department of Surgery, Brigham and Women’s Hospital, Boston, Massachusetts, United States of America; 4 Harvard School of Dental Medicine, Boston, Massachusetts, United States of America; 5 Division of Plastic and Reconstructive Surgery, Department of Surgery, Brigham and Women’s Hospital, Boston, Massachusetts, United States of America; Örebro University, SWEDEN

## Abstract

**Background:**

Surgical interventions are being increasingly recognized as cost-effective global priorities, the utility of which are frequently measured using either quality-adjusted (QALY) or disability-adjusted (DALY) life years. The objectives of this study were to: (1) identify surgical cost-effectiveness studies that utilized a formulation of the QALY or DALY as a summary measure, (2) report on global patterns of QALY and DALY use in surgery and the income characteristics of the countries and/or regions involved, and (3) assess for possible associations between national/regional-income levels and the relative prominence of either measure.

**Study Design:**

PRISMA-guided systematic review of surgical cost-effectiveness studies indexed in PubMed or EMBASE prior to December 15, 2014, that used the DALY and/or QALY as a summary measure. National locations were used to classify publications based on the 2014 World Bank income stratification scheme into: low-, lower-middle-, upper-middle-, or high-income countries. Differences in QALY/DALY use were considered by income level as well as for differences in geographic location and year using descriptive statistics (two-sided Chi-squared tests, Fischer’s exact tests in cell counts <5).

**Results:**

A total of 540 publications from 128 countries met inclusion criteria, representing 825 “national studies” (regional publications included data from multiple countries). Data for 69.0% (569/825) were reported using QALYs (2.1% low-, 1.2% lower-middle-, 4.4% upper-middle-, and 92.3% high-income countries), compared to 31.0% (256/825) reported using DALYs (46.9% low-, 31.6% lower-middle-, 16.8% upper-middle-, and 4.7% high-income countries) (p<0.001). Studies from the US and the UK dominated the total number of QALY studies (49.9%) and were themselves almost exclusively QALY-based. DALY use, in contrast, was the most common in Africa and Asia. While prominent published use of QALYs (1990s) in surgical cost-effectiveness studies began approximately 10 years earlier than DALYs (2000s), the use of both measures continues to increase.

**Conclusion:**

As global prioritization of surgical interventions gains prominence, it will be important to consider the comparative implications of summary measure use. The results of this study demonstrate significant income- and geographic-based differences in the preferential utilization of the QALY and DALY for surgical cost-effectiveness studies. Such regional variation holds important implications for efforts to interpret and utilize global health policy research. **PROSPERO registration number**: CRD42015015991

## Introduction

Worldwide, more than 5 billion people live without access to surgical care, according to recent estimates from the Lancet Commission on Global Surgery[1], and according to the third edition of the Disease Control Priorities[2], more than 1.5 million preventable deaths are related to surgical conditions each year. Ongoing efforts to assess the cost-effectiveness of surgical interventions further reveal that in addition to saving lives, the quality of the lives saved are also improved.[2, 3] Scaling-up of surgical interventions in low- and middle-income countries (LMIC) is often as cost effective as more widely-recognized interventions such as vitamin A provision or the promotion of vaccine use. [3, 4] Given that surgical interventions account for a greater global disease burden than that of tuberculosis, HIV/AIDS, and malaria combined, [3] it has been argued that the ability to provide needed surgical care constitutes an essential part of a functional health system. [1, 2]

Careful consideration of the cost-effectiveness of surgical procedures and the related disease burdens that the interventions address is required to prioritize surgical interventions in parallel with the continued development of non-surgical care.[4] Summary measures of population health combine information on mortality and non-fatal health outcomes to provide a mechanism to compare healthcare delivery.[5, 6] These measures can offer an overarching quantitative perspective of a population’s wellbeing while serving three primary functions: (1) compare population health across communities and over time; (2) provide an overall picture of the diseases, injuries, and risk factors that contribute the most to losses or gains in health; and (3) guide assessment of the strengths, weaknesses, and needs of a health (information) system.[1]

A variety of summary measures have emerged. Two of the most common—the quality-adjusted life year (QALY) and disability-adjusted life year (DALY)–rose to prominence among economic cost-effectiveness analyses and global disease prioritization [most notably the World Health Organization (WHO) Global Burden of Disease (GBD) and successor Generalized Cost-Effectiveness Analysis (GCEA)], respectively.[7, 8] The modern QALY was first used by Zeckhauser and Shepard[9] in 1976 as a measure intended to combine the duration and quality of a person’s life. It has become widely accepted as a reference standard in many cost-effectiveness analyses,[8, 10, 11] despite continuing debate regarding its theoretical assumptions, consistency of calculation, and practical implications. Following the emergence of the QALY and formalization of its more modern conceptualization,[10, 11] the DALY emerged in the late 1980s and early 1990s as a measure intended to calculate disease burdens by considering both years of life lost (YLL) and years lived with disability (YLD) (Table 1). Most formulations include some form of population-based disability weighting; some incorporate age-based “social” weighting. Table 1 provides a brief outline of the characteristics and potential differences in outcome between the QALY and DALY.[1, 7, 8, 11–23] Much like the QALY, the DALY has also gained popularity as a cost-effectiveness measure due, in large part, to the support of organizations such as the WHO and World Bank.[7, 8]

**Table 1:** Brief overview of characteristics and potential differences in outcome between the QALY and DALY families of summary measures.

Characteristics	DALY	QALY	Reference
Year Developed and Definition	1980-90s: YLL from premature mortality + YLD from health conditions	1970s: life-years weighted by quality, accumulated over time	[1, 11–13]
Main Usage	Provide summary measures on disease burden to track changes in population health over time; emerging utility in economic evaluation	Provide summary measures of health program outcomes for economic evaluation and resource allocation	[1, 11]
Endorsers	The World Health Organization and the World Bank	The (UK) National Institute for Health and Care Excellence (NICE) and the Panel on Cost-Effectiveness in Health and Medicine	[14, 15]
Perspective	Health loss from theoretical life expectancy; based on disability	Health gain accumulated (no theoretical life expectancy); based on quality	[16, 17]
Health Construct	One's disability and capacity to function across multiple domains	One's trajectory through value-adjusted health states over time	[1, 16–18]
Weights	Disability weights: 0 (perfect health) to 1 (death); no interval properties. Can incorporate age-based “social weights”	Utility scores: 0 (death) to 1 (perfect health); with interval properties	[17, 18]
Data Source	Representative general population	Patients, experts, target population	[17, 19]
**Quantitative Differences**	DALYs averted can be less than QALYs gained depending on age-weighting,* discounting, intervention, disability		[7]
	QALYs gained may exceed or fall behind DALYs averted depending on age* and life expectancy		[8]
	Both QALY and DALY give more weight to youth		[20]
	Observation that QALY is more likely used for non-communicable diseases, whereas DALY's position is unclear		[19]
	Predicted QALYs gained were larger than DALYs averted for a single vaccination program		[21]
	Methodologies relying on disability weights neglect certain surgical conditions		[22]
	QALY and DALY are equivalent with fixed reference age; without this, interventions for the elderly can increase the burden		[7]
	DALYs increase for conditions with long-term disability and for conditions with a high probability of successful treatment		[23]

The top half of this table is a side-by-side comparison of the DALY and QALY with regard to characteristics listed on the left-most column. The bottom half of this table references studies that have illustrated potential quantitative differences in the calculated DALYs averted and QALYs gained for a given health program.

*Age weighting was not applied in the latest iteration of GBD (2010) Study.

NICE, National Institute for Health and Care Excellence; YLD, years lost due to disability; YLL, years of life lost.

Nevertheless, despite the prominence of both measures in cost-effectiveness analyses, their relative utilization remains poorly understood, particularly as it pertains to surgical prioritization. To this end, the objectives of this study were to: (1) identify surgical cost-effectiveness studies that utilized a formulation of the QALY or DALY as a summary measure, (2) report on global patterns of QALY and DALY use in surgery and the income characteristics of the countries and/or regions involved, and (3) assess for possible associations between national/regional income levels and the relative prominence of either measure.

## Methods

Using a PRISMA-guided approach (PROSPERO protocol registration number: CRD42015015991; S5 File) [22,23], the study identified surgical cost-effectiveness studies indexed in PubMed or EMBASE prior to December 15, 2014 (the date the last search was performed) that used the DALY and/or QALY as a summary measure in identified regional/national/sub-national locations around the globe. For each identified study, the corresponding location was used to classify publications based on the 2014 World Bank income stratification scheme[24] into: low-, lower-middle-, upper-middle-, or high-income countries.

### Information Collection, Source, and Search Strategy

The study’s search strategy was developed by the authors in consultation with a research librarian at Boston University Medical Center Alumni Medical Library. Articles were identified in PubMed and EMBASE electronic databases using MeSH (medical subheadings) and explosive search strategies with variations of the following keywords: *cost* AND *surgery* AND *QALY* AND the names of the 214 World Bank recognized world economies, stratified by income level. An analogous search strategy was repeated with variations of the term *DALY*. In total, four searches were performed for QALY articles (one for each of the four income categories), and four searches were conducted for DALY articles. An example of a specific controlled vocabulary MeSH entry is provided in S1 File. There were no time restrictions for study inclusion; the last search was conducted on December 15, 2014.

**Fig 1:**
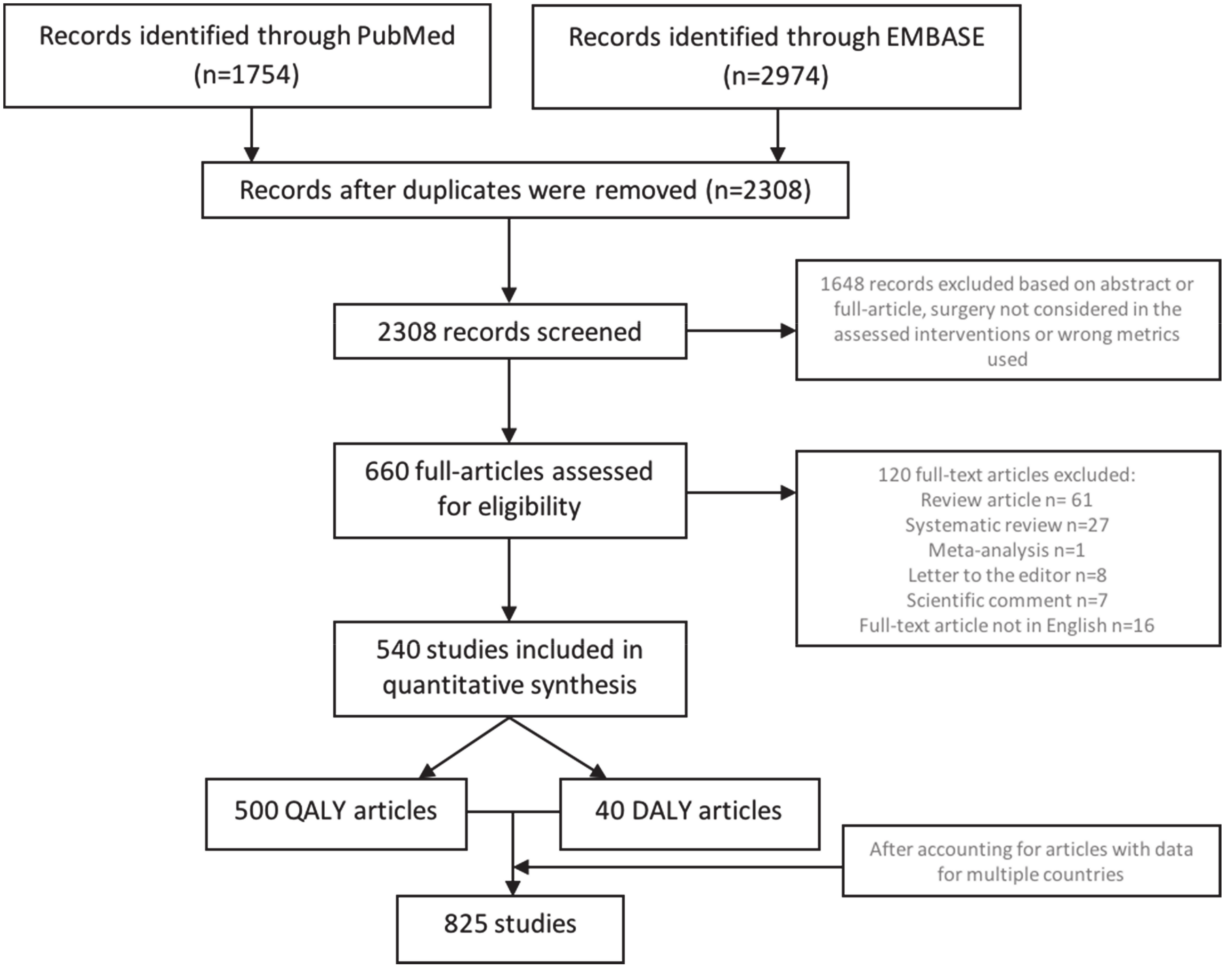
Systematic review selection flow chart.

Two health services researchers independently screened articles for eligibility by reviewing article titles and abstracts. Articles that met the eligibility criteria (defined below and in Fig 1) were recorded based on unique identifiers [*e*.*g*. PubMed ID (PMID), digital object identifier (DOI) and/or title). Articles not identified by both reviewers were discussed between the two reviewers and reevaluated for inclusion or exclusion.

Reviewers collected the following data: (1) metric used (DALY or QALY), (2) country (countries) in which the study outcomes were measured, and (3) year of publication. The number of studies published for low-, lower-middle-, upper-middle-, and high-income countries was tabulated and used as the primary outcome measure. Articles were further stratified by year of publication to account for the gap in timeframe between when the DALY (approx. 1988) and QALY (approx. 1970) were introduced.[9, 12, 13, 25]

### Eligibility Criteria

#### Type of studies

Published primary literature reporting on surgical cost-effectiveness in English with no date restrictions

#### Type of intervention sought

Surgical: The study utilized a modified version of Debas *et al*.’s definition from 2006, defining surgery as an operation performed with the direct goal of treating, prophylactically treating, or curing a person that requires sutures, incisions, excisions, manipulations, or other invasive procedures that usually, but not always, requires local, regional, or general anesthesia.[26] Based on this definition, many diagnostic and bedside procedures were excluded (*e*.*g*. biopsies, endoscopies, colonoscopies, invasive imaging, extracorporeal membrane oxygenation, and dialysis catheter placement). Noninvasive laser procedures like laser trabeculoplasty were also excluded.

#### Outcome measure(s) of included studies

Assessment of the utility of some form of surgical intervention associated with either DALYs averted or QALYs gained

### Statistical analysis

Descriptive statistics (Chi-squared tests; Fisher’s exact tests in cell counts <5) were used to compare the frequency (n) and percentage (%) of studies based on variations in summary measure, countries’ World Bank income level, countries’ geographic location, and year of publication. Two-sided p-values<0.05 were considered significant. All statistical and geographic analyses were performed using SPSS 22 (IBM corporation, Amrok, NY, USA).

## Results

### Article selection process

Of the 4,728 articles identified through PubMed and EMBASE, 2,308 non-duplicate titles and abstracts were screened for eligibility; 660 were retrieved for full-text evaluation. Of these, 120 consisted of secondary literature and were subsequently eliminated, leaving a total of 540 publications included in the analysis (S3 and S4 Files). The majority (n = 500) used a formulation of the QALY; 40 used a formulation of the DALY (Fig 1). Data on surgical interventions reported in the 540 studies represented work located in 128 countries. To account for publications reporting data for multiple countries, a tally of individual country-based cost-effectiveness estimates was used, bringing the analytical total of country-based QALY and DALY estimates to 825 “national studies.” A dataset of included information is available as supporting information online.

### Use of QALYs and DALYs in surgical cost-effectiveness studies by income level

**Fig 2:**
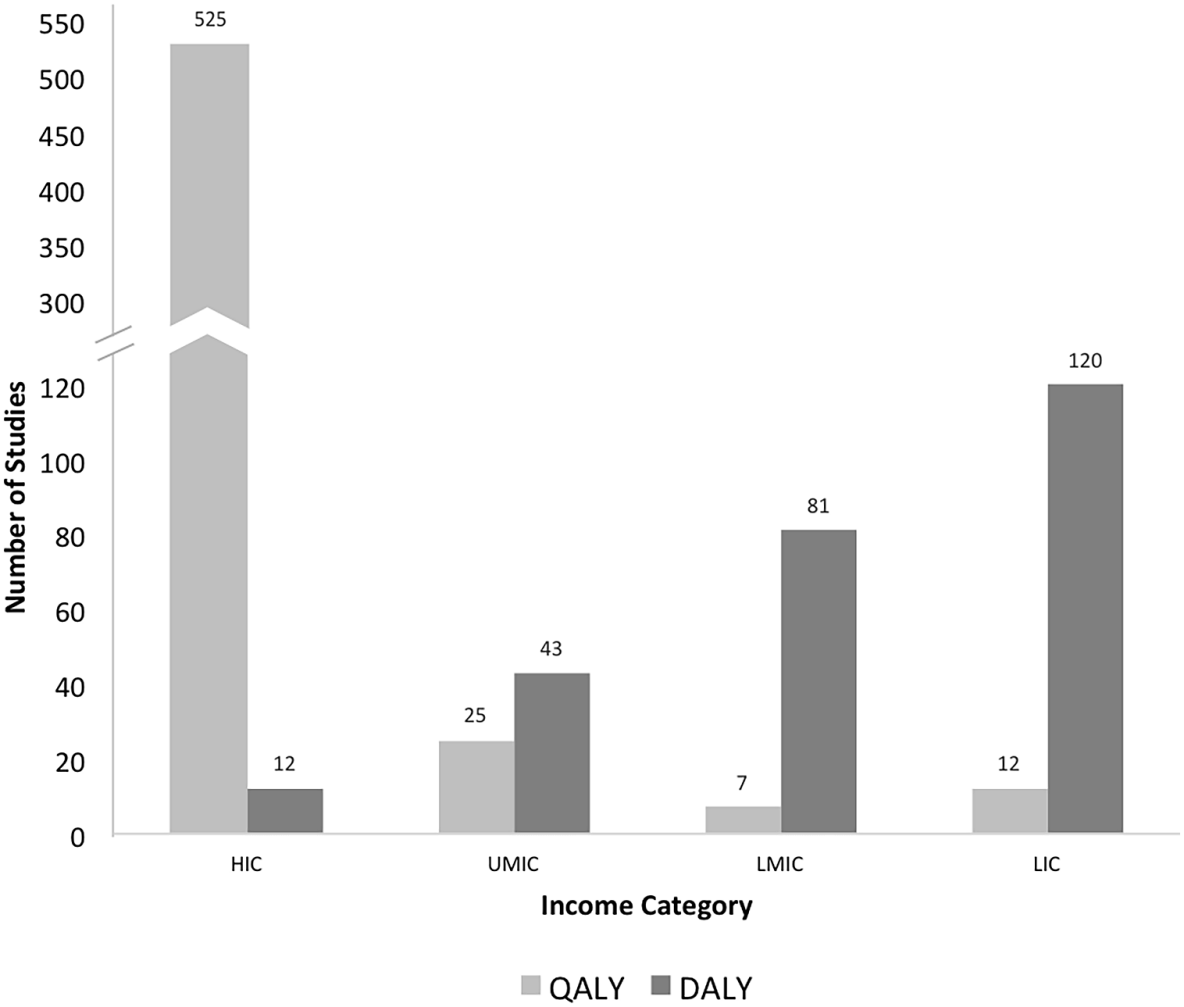
Number of national surgical cost-effectiveness studies using the QALY or DALY metric by World Bank national income level (n = 825). Graph shows the number of surgical cost-effectiveness QALY and DALY-based studies (y-axis) published based on work conducted in countries corresponding to the four World-Bank-defined national income levels (x-axis). Abbreviations: DALY, disability-adjusted life year; LIC, low-income country; LMIC, lower-middle-income country; UMIC, upper-middle- income country; HIC, high-income country; QALY, quality-adjusted life year

Of the 825 national studies, 69.0% (n = 569) were reported using QALYs: 2.1% in low-, 1.2% in lower-middle-, 4.4% in upper-middle-, and 92.3% in high-income countries. Data for the remaining 31.0% (n = 256) of surgical cost-effectiveness studies were reported using DALYs: 46.9% in low-, 31.6% in lower-middle-, 16.8% in upper-middle-, and 4.7% in high-income countries (Fig 2). Differences in metric use by World Bank national income level were significant (*p*<0.001). Studies in high-income countries comprised the majority of surgical cost-effectiveness analyses (65.1%), and as demonstrated in Fig 2, exhibited a higher QALY:DALY use ratio (44:1) that sharply contrasted the higher DALY:QALY use ratios observed in the lower three income tiers—upper-middle-income: 2:1, lower-middle-income: 12:1, and lower-income: 10:1.

### Global distribution of QALY and DALY use in surgical cost-effectiveness studies

**Fig 3:**
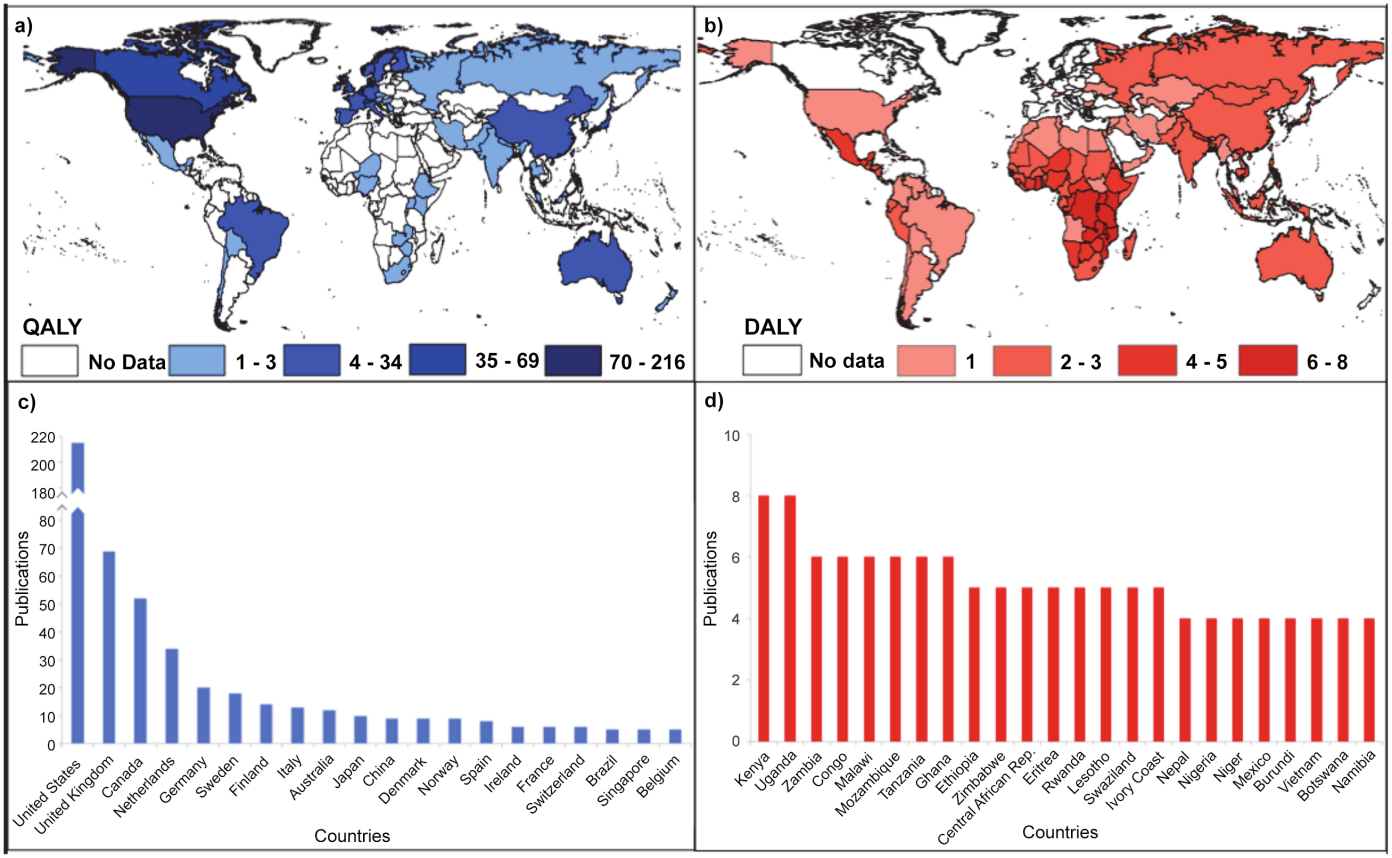
Global distribution of QALY and DALY surgical cost-effectiveness studies. Fig 3a and 3b illustrate the distribution of surgical cost-utility studies using the QALY (blue) and DALY (red) metric, respectively (n = 825). The numbers next to the colored rectangles indicate the range in the number of studies published. Fig 3c and 3d illustrate the countries for which surgical cost-utilities are most frequently reported using the QALY or DALY metric, respectively. Abbreviations: DALY, disability-adjusted life year; QALY, quality-adjusted life year

Density maps presented in Fig 3a (QALY in blue) and Fig 3b (DALY in red) illustrate the global distribution of summary measure use among surgical cost-effectiveness studies. Overall, the United States (US) was the most dense, representing 26.2% of included studies (*n =* 216), followed by the United Kingdom (UK), representing 8.4% (*n* = 69). Studies from the US and UK dominated the total number of QALY-based studies observed (combined 49.9%; n = 284/569) and were themselves almost exclusively QALY-based; one study reported DALY use. In order to dismiss the possibility that inclusion of the US and UK swayed the significance of the association between summary measure use and national income level, we performed a sensitivity analysis excluding these countries and found that the differences remained significant (p<0.001; S2 File).

The five most prolific QALY contributors represented, in descending order, were the US, the UK, Canada, the Netherlands, and Germany (Fig 3c). Non-negligible QALY contributions were also observed throughout most of Europe as well as in Japan, China, Australia, and Brazil (Fig 3a). In striking contrast, the countries that most frequently used the DALY included Kenya and Uganda (tied for the most prolific), followed by Zambia, Congo, Malawi, Mozambique, Tanzania, and Ghana (Fig 3d). DALY studies were most pronounced throughout regions of Africa and Asia and, notably in North America, in the country of Mexico (Fig 3b). The near absence of QALY studies (Fig 3a) throughout much of Africa, Asia, and Central/South America coupled with the correspondingly limited presence of DALY studies (Fig 3b) in high-income countries points to a seemingly stark polarization in DALY versus QALY summary measure use.

### Surgical cost-effectiveness QALY- and DALY-based publications by year

**Fig 4:**
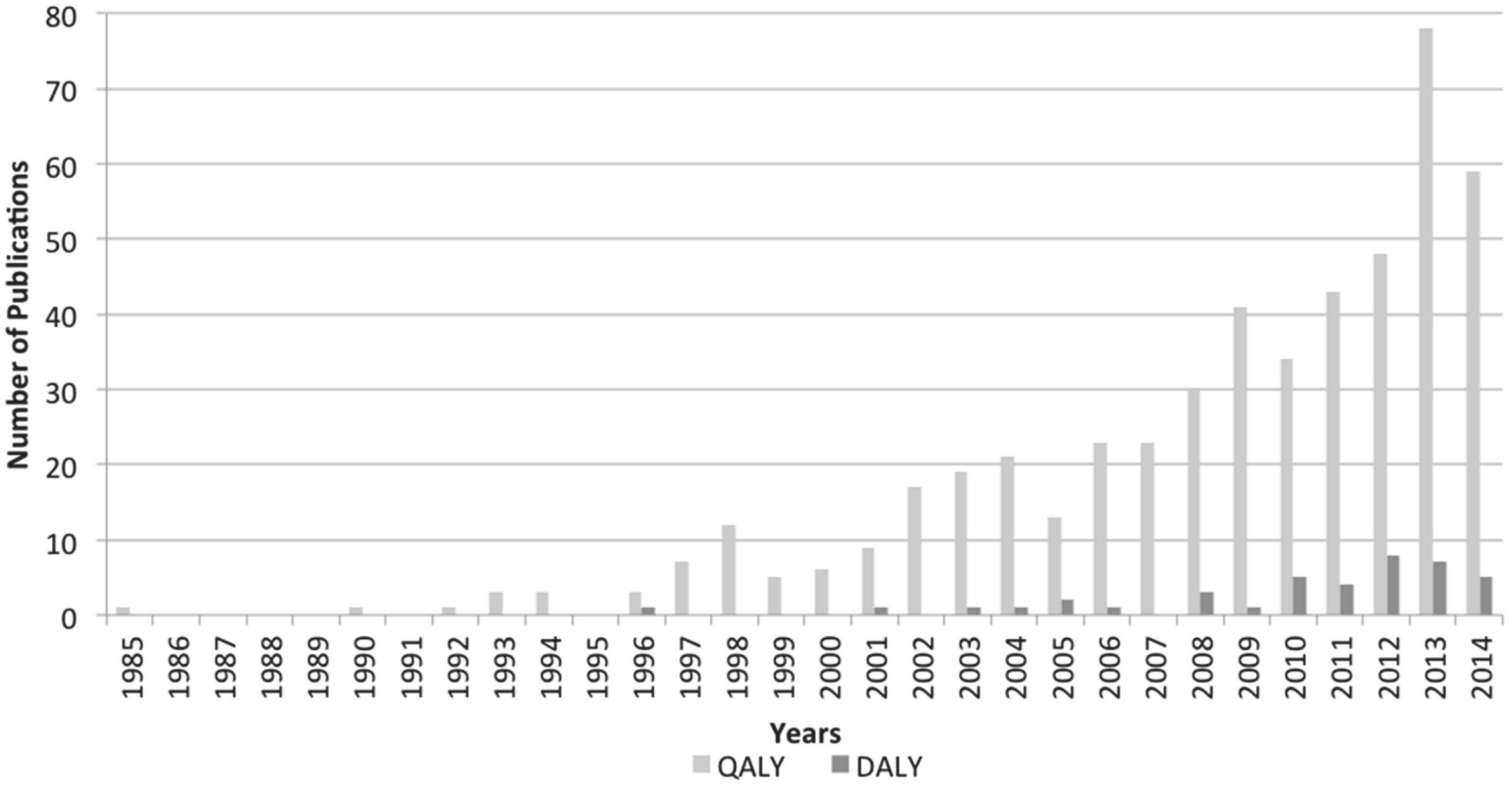
Number of included QALY and DALY publications by year (n = 540). The number of surgical cost-effectiveness publications using the QALY (light gray) has increased beginning in the 1990s. While there is some growth in the use of the DALY (dark gray), its use is not as pronounced as the use of the QALY. The drop-off in literature found for 2014 is likely due to lag in time between publication and entry into PubMed and EMBASE.

The number of QALY and DALY surgical cost-effectiveness studies by year of publication is illustrated in Fig 4. The QALY metric was founded in the 1970s (not shown), and first appeared in press in 1976.[9] Its first included use in the surgical cost-effectiveness literature was published by Williams[27] in 1985. The DALY metric was founded around 1988 with its first included use in the surgical cost-effectiveness literature published by Marseille in 1996 [28]. Included QALY studies began increasing throughout the 1990s, while included DALY studies did not meaningfully increase until after the year 2000. Despite global differences in their timing and dominant geographical distributions, both summary measures, as well as the overall number of surgical cost-effectiveness analyses, have been increasing (p<0.001), especially in recent years.

## Discussion

As global prioritization of surgical interventions gains prominence, it will be important to consider the comparative implications of summary measure use. This study compared trends in the use of formulations of the DALY and QALY as summary measures in published surgical cost-effectiveness studies. A combined total of 540 publications, representing 825 national studies from 128 countries were included and used to assess variations in preferential summary measure use over time, by geographic location, and among World Bank-defined income strata. The results revealed that surgical cost-effectiveness studies involving lower-income countries more frequently employed DALYs, whereas studies involving higher-income countries were more likely to use QALYs. Geographic differences, depicted in Fig 3a and 3b, demonstrate a similar trend with the greatest number of DALY-based studies coming from countries in Africa and Asia, such as Kenya and Uganda (the most frequent two), relative to the nearly 50% of QALY-based studies conducted in the US and UK. Such difference points to a seemingly stark polarization in DALY versus QALY summary measure use that may have important implications for the growing international focus on efforts to interpret and utilize global health policy research. [1–5]

One area where their influence may have a direct effect is in the utilization of published research to influence resource allocation on a regional, national, or sub-national scale. In a comprehensive analysis contrasting the benefit of an intervention as a health gain (using QALYs) or disability reduction (using DALYs), Airoldi *et al*. demonstrated how health planners could rank health interventions in a systematically different way depending on the summary measure used, even when assumptions about costs and effectiveness were the same and when health and disability weights were made consistent.[7] Health economists have shown that, under certain circumstances, DALYs (which are better when small—years of healthy life lost) can increase or yield a “statistically greater burden” for an intervention that prevents mortality—while the years of life lost will decrease, the years lived with disability may be substantially greater in the wake of increased survival—leading to an overall worse summary measure.[8. 9] As a result, DALY use may tend to favor surgical interventions that address conditions with improved survival but which lead to longer-term disability over those with higher mortality.[5, 6] They are, however, consistent across a population. Formulations of the QALY, in contrast, are designed to be individually value-based. It has been argued that the impact of interventions on disease burdens may be more adequately captured by value-adjusted parameters inherent in the QALY.[18]. Authors like Weinstein *et al*. suggest that the use of QALY (which is better when large—years of healthy life gained) enables researchers to account for the fact that disability is not always a hallmark of a high-priority health state and that health states may be better accounted for in terms of the “value added” by their treatment.[17]

Historical motivations, including economic-based patient-decision analyses in higher-income countries like the UK and US where the QALY is prominent [9–12] versus DALY-based geographic needs assessments conducted by the WHO and World Bank [8, 9], inevitably also come into play. Despite considerable rhetoric and stark differences in income- and geographic-based utilization demonstrated by the present study, there remains no concrete evidence of one summary measure being “better” than the other. Both provide a quantitative model [5–7], neither without flaws.[29] As the field of global surgery continues to develop, further studies will be warranted to determine the extent to which there is a difference between summary measure predictions for interventions deemed “cost effective.”

Our results are consistent with previous studies that have sought to identify surgical cost-effectiveness interventions assessed in lower-resource settings. Like the work of Chao *et al*. [3] and Grimes *et al*. [4] intended to assess the cost-effectiveness of specific surgical interventions, the results of our assessment demonstrate a relative abundance of DALY literature in lower-income settings and a comparative lack of QALY studies. However, regardless of the metric used, it is important to note that only 16.0% (*n =* 132) of the 825 national studies were conducted in World Bank-defined low-income countries; an additional 10.7% (*n* = 88) were conducted in lower-middle-income countries.

The relative dearth of surgical cost-effectiveness information in these lower-income countries with the greatest unmet surgical need [1, 2] poses a problem for resource allocation. Decision-makers often cannot make informed decisions regarding the cost and benefits of surgical interventions, especially in the context of competing disease priorities, limited resources, and considerable disease burdens with which to contend. Beyond policy prioritization, the lack of surgical cost-effectiveness data in lower-resource countries also reflects several broader issues. For example, in many lower-income settings, there is a lack of surgical capacity needed to ascertain the effectiveness of a surgical intervention. This limited availability of resources includes operating rooms, trained physicians, trained anesthesiologists, and surgical equipment.[30] Modeling can, theoretically, impute values for this missing data, but for countries like Rwanda with fewer than 50 trained surgeons for 10.6 million people, the underlying lack of infrastructure makes the conclusions both suspect and nearly impossible to interpret.[31] Lower-resource settings may also not have sufficient data to carry out such studies, even when adequate surgical infrastructure is in place. In order to gather the information needed for cost-effectiveness analyses, one needs to consider the acquisition of procedural cost data, indirect cost data, utility scores for the target population, and complications of procedures, none of which are readily available in countries that have not transitioned towards more refined methods of record-keeping. Locating the bottleneck in efforts to mobilize surgical care may prove an important starting point to begin addressing this issue.

The present study is not without its limitations. Restricting searches of published articles to two prominent databases of English-language publications enabled standardization of the search parameters, but may have excluded studies conducted by non-English speaker researchers or which were published in local journals. The majority of lower-resource countries speak a non-English primary language, and the extent to which relevant studies may have been published in other languages remains unknown. The search criteria were, however, sufficiently broad to identify and screen over 2,300 non-duplicated “surgical cost-effectiveness” studies. Articles that made no mention of their country of origin may also have been missed. Consideration of author origin as a supplement to a non-specified study setting (where reasonable to do so) helped to greatly reduce this limitation. Lack of time constraints on study inclusion may have biased the results in favor of the QALY measure, as the DALY was not introduced until several years later. However, despite this concern, a delay in DALY use among surgical cost-effectiveness studies does not appear to be particularly pronounced. Our study identified only one included QALY publication prior to the DALY’s establishment in the surgical cost-effectiveness literature (4), and while it was not until 2003 that the authors of the GBD made recommendations regarding how to modify the DALY for use in cost-effectiveness analyses,[32] insubstantial numbers of studies using either metric were published prior to the year 2000 (36 QALY publications and 1 DALY publication) when compared with the total number of included surgical cost-effectiveness publications identified (*n* = 540). The study relied on published literature and was not able to consider unpublished health assessments used and conduced by non-governmental organizations and/or ministries of health for the expressed purpose of resource allocation and priority setting, many of which may be DALY-based. Lastly, while the results of the study indicate a stark difference in the income- and geographic-distributions of surgical cost-effectives research related to summary measure use, it is important to understand that the differential use of the two metrics may be influenced as much, if not more, by historical lineage than by any sort of preferential decision on the part of researchers. The DALY was developed by the WHO for use in assessment of developing settings [7, 8, 33], while the QALY was founded in the UK and has since risen to prominence in much of the Western world.[1]

## Conclusion

Differences in the distribution of QALY and DALY summary measure use among published surgical cost-effectiveness assessments exist and are closely associated with both the location and income of the country where the analysis is conducted. There is a predominant use of the QALY in studies involving higher-income countries and the DALY in studies involving lower-income countries. The dichotomy in metrics illustrates an important distinction between cost-effectiveness considerations conducted in different settings. Whether a result of preferential use decisions or historical familiarity and experience, differences in interpretations between the two metrics (Table 1) need to be carefully weighed, especially as the global prioritization of “cost-effective” surgical interventions continues to increase in coming years. Summary measures of population health provide a quantitative means of capturing the experience of a population. Significant regional variation in their use, as demonstrated in this study, holds important implications for growing efforts to interpret and utilize global health policy research.

## Supporting Information

S1 File Sample PubMed search term. (DOCX)

S2 File Sensitivity analysis assessing the significance of the association between summary measure use and national income level when the United States and the United Kingdom were excluded. (DOCX)

S3 File QALY and DALY Surgical Cost-Utility Analyses publications included. (DOCX)

S4 File **Table A.** Minimal dataset of publications included in the analysis along with the year of publication, country/countries involved, and cost-utility analysis summary measure and income-level group. **Table B.** Summary of publications included in the analysis by country involved. **Table C.** Summary of publications included in the analysis by cost-utility analysis measure used.
(XLSX)

S5 File Supporting Information. Checklist of items included in the systematic review according to PRISMA guidelines.
(DOC)
