# Supplementary material for: Global Patterns of QALY and DALY Use in Surgical Cost-Utility Analyses: A Systematic Review
Source: PLoS One. 2016 Feb 10;11(2):e0148304. doi: 10.1371/journal.pone.0148304 (PMC4749322; doi:10.1371/journal.pone.0148304)
Supplement: S1 File — (DOCX) [file pone.0148304.s001.docx]

**S1 File.** Sample PubMed search term

("surgery"[Subheading] OR "Surgical Procedures, Operative"[Mesh] OR "surgery" OR "surgeries" OR "surgical" OR "surgical intervention*" OR "surgical condition*" OR "surgical disease*") AND ("Cost-Benefit Analysis"[Mesh] OR "Costs and Cost Analysis"[Mesh] OR "Cost of Illness"[Mesh] OR "Health Care Costs"[Mesh] OR "Economics"[Mesh] OR "cost utility analysis" OR "cost-utility analysis" OR "CUA" OR "CEA" OR "CBA") AND ("Afghanistan"[Mesh] OR "Bangladesh"[Mesh] OR "Benin"[Mesh] OR "Burkina Faso"[Mesh] OR "Burundi"[Mesh] OR "Cambodia"[Mesh] OR "Central African Republic"[Mesh] OR "Chad"[Mesh] OR "Comoros"[Mesh] OR "Democratic Republic of the Congo"[Mesh] OR "Eritrea"[Mesh] OR "Ethiopia"[Mesh] OR "Gambia"[Mesh] OR "Guinea"[Mesh] OR "Guinea-Bissau"[Mesh] OR "Haiti"[Mesh] OR "Kenya"[Mesh] OR "Democratic People's Republic of Korea"[Mesh] OR "Liberia"[Mesh] OR "Madagascar"[Mesh] OR "Malawi"[Mesh] OR "Mali"[Mesh] OR "Mozambique"[Mesh] OR "Myanmar"[Mesh] OR "Nepal"[Mesh] OR "Niger"[Mesh] OR "Rwanda"[Mesh] OR "Sierra Leone"[Mesh] OR "Somalia"[Mesh] OR "Tajikistan"[Mesh] OR "Tanzania"[Mesh] OR "Togo"[Mesh] OR "Uganda"[Mesh] OR "Zimbabwe"[Mesh]) AND ("Disability-Adjusted Life Years" OR "DALY*" OR "disability adjusted life year*" OR "disability-adjusted life year*")
