# Supplementary material for: Global Patterns of QALY and DALY Use in Surgical Cost-Utility Analyses: A Systematic Review
Source: PLoS One. 2016 Feb 10;11(2):e0148304. doi: 10.1371/journal.pone.0148304 (PMC4749322; doi:10.1371/journal.pone.0148304)
Supplement: S2 File — (DOCX) [file pone.0148304.s002.docx]

**S2 File.** Sensitivity analysis

Summary of QALY and DALY CUA studies classified by countries’ level of income where the study was conducted (Original Data, *n*=825)

|  |  | **Type of CUA study** | |
| --- | --- | --- | --- |
|  |  | QALY | DALY |
| **Income Level** | HIC | 525 | 12 |
|  | UMIC | 25 | 43 |
|  | LMIC | 7 | 81 |
|  | LIC | 12 | 120 |

χ^2^ *p*<0.001

Summary of QALY and DALY CUA studies classified by countries’ level of income where the study was conducted (Excluding US and UK studies, *n*=540)

|  |  | **Type of CUA study** | |
| --- | --- | --- | --- |
|  |  | QALY | DALY |
| **Income Level** | HIC | 240 | 12 |
|  | UMIC | 25 | 43 |
|  | LMIC | 7 | 81 |
|  | LIC | 12 | 120 |

χ^2^ *p*<0.001

Abbreviations: CUA= Cost-Utility Analysis; HIC= High-Income Country; UMIC= Upper-Middle-Income Country; LMIC= Lower-Middle-Income Country; LIC= Low-Income Country; US= United States; UK= United Kingdom
