# Supplementary material for: Global Patterns of QALY and DALY Use in Surgical Cost-Utility Analyses: A Systematic Review
Source: PLoS One. 2016 Feb 10;11(2):e0148304. doi: 10.1371/journal.pone.0148304 (PMC4749322; doi:10.1371/journal.pone.0148304)
Supplement: S3 File — (DOCX) [file pone.0148304.s003.docx]

**S3 File.** QALY and DALY Surgical Cost-Utility Analyses publications included.

1. Cost-utility analysis of open versus laparoscopic groin hernia repair: results from a multicentre randomized clinical trial. Br J Surg. 05/ 2001;88(5):653-661.

2. Criteria of candidacy for unilateral cochlear implantation in postlingually deafened adults II: cost-effectiveness analysis. Ear Hear. 08/ 2004;25(4):336-360.

3. Abbott DE, Merkow RP, Cantor SB, et al. Cost-effectiveness of treatment strategies for pancreatic head adenocarcinoma and potential opportunities for improvement. Ann Surg Oncol. 11/ 2012;19(12):3659-3667.

4. Abbott DE, Tzeng C-WD, Merkow RP, et al. The cost-effectiveness of neoadjuvant chemoradiation is superior to a surgery-first approach in the treatment of pancreatic head adenocarcinoma. Ann Surg Oncol. 12/ 2013;20 Suppl 3:500-508.

5. Abell RG, Vote BJ. Cost-effectiveness of femtosecond laser-assisted cataract surgery versus phacoemulsification cataract surgery. Ophthalmology. 01/ 2014;121(1):10-16.

6. Åberg F, Maklin S, Rasanen P, et al. Cost of a quality-adjusted life year in liver transplantation: the influence of the indication and the model for end-stage liver disease score. Liver Transpl. 11/ 2011;17(11):1333-1343.

7. Achelrod D, Stargardt T. Cost-utility analysis comparing heavy-weight and light-weight mesh in laparoscopic surgery for unilateral inguinal hernias. Appl Health Econ Health Policy. 04/ 2014;12(2):151-163.

8. Adam T, Lim SS, Mehta S, et al. Cost effectiveness analysis of strategies for maternal and neonatal health in developing countries. BMJ. 11/12 2005;331(7525):1107-1107.

9. Al MJ, Koopmanschap MA, van Enckevort PJ, et al. Cost-effectiveness of lung transplantation in The Netherlands: a scenario analysis. Chest. 01/ 1998;113(1):124-130.

10. Alali AS, Naimark DMJ, Wilson JR, et al. Economic evaluation of decompressive craniectomy versus barbiturate coma for refractory intracranial hypertension following traumatic brain injury. Crit Care Med. 10/ 2014;42(10):2235-2243.

11. Alkire BC, Vincent JR, Burns CT, Metzler IS, Farmer PE, Meara JG. Obstructed labor and caesarean delivery: the cost and benefit of surgical intervention. PLoS One. / 2012;7(4).

12. Ament JD, Greene KR, Flores I, et al. Health impact and economic analysis of NGO-supported neurosurgery in Bolivia. J Neurosurg Spine. 04/ 2014;20(4):436-442.

13. Amin AP, Reynolds MR, Lei Y, et al. Cost-effectiveness of everolimus- versus paclitaxel-eluting stents for patients undergoing percutaneous coronary revascularization (from the SPIRIT-IV Trial). Am J Cardiol. 09/15 2012;110(6):765-770.

14. Ananthapavan J, Moodie M, Haby M, Carter R. Assessing cost-effectiveness in obesity: laparoscopic adjustable gastric banding for severely obese adolescents. Surg Obes Relat Dis. / 2010;6(4):377-385.

15. Anderson J, Wilson D, Templeton DJ, Grulich A, Carter R, Kaldor J. Cost-effectiveness of adult circumcision in a resource-rich setting for HIV prevention among men who have sex with men. J Infect Dis. 12/15 2009;200(12):1803-1812.

16. Anselmino M, Bammer T, Fernandez Cebrian JM, Daoud F, Romagnoli G, Torres A. Cost-effectiveness and budget impact of obesity surgery in patients with type 2 diabetes in three European countries(II). Obes Surg. 11/ 2009;19(11):1542-1549.

17. Anyanwu AC, McGuire A, Rogers CA, Murday AJ. An economic evaluation of lung transplantation. J Thorac Cardiovasc Surg. 03/ 2002;123(3):411-418.

18. Araujo CDM, Veiga DF, Hochman BS, et al. Cost-utility of reduction mammaplasty assessed for the Brazilian public health system. Aesthet Surg J. 11/ 2014;34(8):1198-1204.

19. Axelrod DA, Gheorghian A, Schnitzler MA, et al. The economic implications of broader sharing of liver allografts. Am J Transplant. 04/ 2011;11(4):798-807.

20. Azuara-Blanco A, Burr JM, Cochran C, et al. The effectiveness of early lens extraction with intraocular lens implantation for the treatment of primary angle-closure glaucoma (EAGLE): study protocol for a randomized controlled trial. Trials. / 2011;12:133-133.

21. Bakhai A, Stone GW, Grines CL, et al. Cost-effectiveness of coronary stenting and abciximab for patients with acute myocardial infarction: results from the CADILLAC (Controlled Abciximab and Device Investigation to Lower Late Angioplasty Complications) trial. Circulation. 12/09 2003;108(23):2857-2863.

22. Bakhai A, Stone GW, Mahoney E, et al. Cost effectiveness of paclitaxel-eluting stents for patients undergoing percutaneous coronary revascularization: results from the TAXUS-IV Trial. J Am Coll Cardiol. 07/18 2006;48(2):253-261.

23. Baltussen R, Smith A. Cost effectiveness of strategies to combat vision and hearing loss in sub-Saharan Africa and South East Asia: mathematical modelling study. BMJ. / 2012;344.

24. Baltussen R, Sylla M, Mariotti SP. Cost-effectiveness analysis of cataract surgery: a global and regional analysis. Bull World Health Organ. 05/ 2004;82(5):338-345.

25. Baltussen RMPM, Sylla M, Frick KD, Mariotti SP. Cost-effectiveness of trachoma control in seven world regions. Ophthalmic Epidemiol. 04/ 2005;12(2):91-9101.

26. Barnieh L, Gill JS, Klarenbach S, Manns BJ. The cost-effectiveness of using payment to increase living donor kidneys for transplantation. Clin J Am Soc Nephrol. 12/ 2013;8(12):2165-2173.

27. Barton GR, Stacey PC, Fortnum HM, Summerfield AQ. Hearing-impaired children in the United Kingdom, IV: cost-effectiveness of pediatric cochlear implantation. Ear Hear. 10/ 2006;27(5):575-588.

28. Bastos Filho R, Lermontov S, Borojevic R, Schott PC, Gameiro VS, Granjeiro JM. Cell therapy of pseudarthrosis. Acta Ortop Bras. / 2012;20(5):270-273.

29. Beard JH, Oresanya LB, Ohene-Yeboah M, Dicker RA, Harris HW. Characterizing the global burden of surgical disease: a method to estimate inguinal hernia epidemiology in Ghana. World J Surg. 03/ 2013;37(3):498-503.

30. Beauchamp CL, Beauchamp GR, Stager DR, Brown MM, Brown GC, Felius J. The cost utility of strabismus surgery in adults. J AAPOS. 10/ 2006;10(5):394-399.

31. Beauchemin C, Brunette I, Boisjoly H, Freeman EE, Popescu M, Lachaine J. Economic impact of the advent of posterior lamellar keratoplasty in Montreal, Quebec. Can J Ophthalmol. 06/ 2010;45(3):243-251.

32. Beckwith J, Nyman JA, Flanagan B, Schrover R, Schuurman H-J. A health economic analysis of clinical islet transplantation. Clin Transplant. / 2012;26(1):23-33.

33. Bedair H, Cha TD, Hansen VJ. Economic benefit to society at large of total knee arthroplasty in younger patients: a Markov analysis. J Bone Joint Surg Am. 01/15 2014;96(2):119-126.

34. Bischof M, Briel M, Bucher HC, Nordmann A. Cost-effectiveness of drug-eluting stents in a US Medicare setting:a cost-utility analysis with 3-year clinical follow-up data. Value Health. / 2009;12(5):649-656.

35. Blackhouse G, Hopkins R, Bowen JM, et al. A cost-effectiveness model comparing endovascular repair to open surgical repair of abdominal aortic aneurysms in Canada. Value Health. / 2009;12(2):245-252.

36. Boger PC, Turner D, Roderick P, Patel P. A UK-based cost-utility analysis of radiofrequency ablation or oesophagectomy for the management of high-grade dysplasia in Barrett's oesophagus. Aliment Pharmacol Ther. 12/ 2010;32(11-12):1332-1342.

37. Bohmer E, Kristiansen IS, Arnesen H, Halvorsen S. Health and cost consequences of early versus late invasive strategy after thrombolysis for acute myocardial infarction. Eur J Cardiovasc Prev Rehabil. 10/ 2011;18(5):717-723.

38. Bojke L, Hornby E, Sculpher M. A comparison of the cost effectiveness of pharmacotherapy or surgery (laparoscopic fundoplication) in the treatment of GORD. Pharmacoeconomics. / 2007;25(10):829-841.

39. Bosch JL, Beinfeld MT, Muller JE, Brady T, Gazelle GS. A cost-effectiveness analysis of a hypothetical catheter-based strategy for the detection and treatment of vulnerable coronary plaques with drug-eluting stents. J Interv Cardiol. 10/ 2005;18(5):339-349.

40. Bosch JL, Haaring C, Meyerovitz MF, Cullen KA, Hunink MG. Cost-effectiveness of percutaneous treatment of iliac artery occlusive disease in the United States. AJR Am J Roentgenol. 08/ 2000;175(2):517-521.

41. Bosch JL, Kaufman JA, Beinfeld MT, Adriaensen MEAPM, Brewster DC, Gazelle GS. Abdominal aortic aneurysms: cost-effectiveness of elective endovascular and open surgical repair. Radiology. 11/ 2002;225(2):337-344.

42. Bosch JL, Tetteroo E, Mali WP, Hunink MG. Iliac arterial occlusive disease: cost-effectiveness analysis of stent placement versus percutaneous transluminal angioplasty. Dutch Iliac Stent Trial Study Group. Radiology. 09/ 1998;208(3):641-648.

43. Bose S, Ang M, Mehta JS, Tan DT, Finkelstein E. Cost-effectiveness of Descemet's stripping endothelial keratoplasty versus penetrating keratoplasty. Ophthalmology. 03/ 2013;120(3):464-470.

44. Bouvy JC, Fransen PSS, Baeten SA, Koopmanschap MA, Niessen LW, Dippel DWJ. Cost-effectiveness of two endovascular treatment strategies vs intravenous thrombolysis. Acta Neurol Scand. 05/ 2013;127(5):351-359.

45. Bowen JM, Snead OC, Chandra K, Blackhouse G, Goeree R. Epilepsy care in ontario: an economic analysis of increasing access to epilepsy surgery. Ont Health Technol Assess Ser. / 2012;12(18):1-41.

46. Bowen JM, Whelan JP, Hopkins RB, et al. Photoselective vaporization for the treatment of benign prostatic hyperplasia. Ontario Health Technology Assessment Series. 2013;13(2).

47. Boyer TD, Henderson JM, Heerey AM, et al. Cost of preventing variceal rebleeding with transjugular intrahepatic portal systemic shunt and distal splenorenal shunt. J Hepatol. 03/ 2008;48(3):407-414.

48. Boyers D, Kilonzo M, Mostafa A, Abdel-Fattah M. Comparison of an adjustable anchored single-incision mini-sling, Ajust(®) , with a standard mid-urethral sling, TVT-O(TM) : a health economic evaluation. BJU Int. 12/ 2013;112(8):1169-1177.

49. Breitscheidel L. Cost utility of allogeneic stem cell transplantation with matched unrelated donor versus treatment with imatinib for adult patients with newly diagnosed chronic myeloid leukaemia. J Med Econ. / 2008;11(4):571-584.

50. Briggs A, Sculpher M, Britton A, Murray D, Fitzpatrick R. The costs and benefits of primary total hip replacement. How likely are new prostheses to be cost-effective? Int J Technol Assess Health Care. / 1998;14(4):743-761.

51. Briggs A, Sculpher M, Dawson J, Fitzpatrick R, Murray D, Malchau H. The use of probabilistic decision models in technology assessment : the case of total hip replacement. Appl Health Econ Health Policy. / 2004;3(2):79-89.

52. Brosa M, Munoz-Duyos A, Navarro-Luna A, et al. Cost-effectiveness analysis of sacral neuromodulation (SNM) with Interstim for fecal incontinence patients in Spain. Curr Med Res Opin. 03/ 2008;24(3):907-918.

53. Brown GC, Brown MM, Menezes A, Busbee BG, Lieske HB, Lieske PA. Cataract surgery cost utility revisited in 2012: a new economic paradigm. Ophthalmology. 12/ 2013;120(12):2367-2376.

54. Brown GC, Brown MM, Sharma S, Busbee B, Landy J. A cost-utility analysis of interventions for severe proliferative vitreoretinopathy. Am J Ophthalmol. 03/ 2002;133(3):365-372.

55. Brown KL, Wray J, Wood TL, Mc Mahon AM, Burch M, Cairns J. Cost utility evaluation of extracorporeal membrane oxygenation as a bridge to transplant for children with end-stage heart failure due to dilated cardiomyopathy. J Heart Lung Transplant. 01/ 2009;28(1):32-38.

56. Brunner-La Rocca HP, Kaiser C, Bernheim A, et al. Cost-effectiveness of drug-eluting stents in patients at high or low risk of major cardiac events in the Basel Stent KostenEffektivitats Trial (BASKET): an 18-month analysis. Lancet. 11/03 2007;370(9598):1552-1559.

57. Burfeind WR, Jaik NP, Villamizar N, Toloza EM, Harpole DH, D'Amico TA. A cost-minimisation analysis of lobectomy: thoracoscopic versus posterolateral thoracotomy. Eur J Cardiothorac Surg. 04/ 2010;37(4):827-832.

58. Busbee BG, Brown MM, Brown GC, Sharma S. Incremental cost-effectiveness of initial cataract surgery. Ophthalmology. 03/ 2002;109(3):606-612.

59. Busbee BG, Brown MM, Brown GC, Sharma S. Cost-utility analysis of cataract surgery in the second eye. Ophthalmology. 12/ 2003;110(12):2310-2317.

60. Cain-Nielsen AH, Moriarty JP, Stewart EA, Borah BJ. Cost-effectiveness of uterine-preserving procedures for the treatment of uterine fibroid symptoms in the USA. J Comp Eff Res. 09/ 2014;3(5):503-514.

61. Cameron HL, Bernard LM, Garmo VS, Hernandez JB, Asgar AW. A Canadian cost-effectiveness analysis of transcatheter mitral valve repair with the MitraClip system in high surgical risk patients with significant mitral regurgitation. J Med Econ. 08/ 2014;17(8):599-615.

62. Campbell HE, Tait S, Buxton MJ, et al. A UK trial-based cost--utility analysis of transmyocardial laser revascularization compared to continued medical therapy for treatment of refractory angina pectoris. Eur J Cardiothorac Surg. 08/ 2001;20(2):312-318.

63. Campbell HE, Tait S, Sharples LD, et al. Trial-based cost-utility comparison of percutaneous myocardial laser revascularisation and continued medical therapy for treatment of refractory angina pectoris. Eur J Health Econ. 12/ 2005;6(4):288-297.

64. Capomolla S, Febo O, Ceresa M, et al. Cost/utility ratio in chronic heart failure: comparison between heart failure management program delivered by day-hospital and usual care. J Am Coll Cardiol. 10/02 2002;40(7):1259-1266.

65. Caro J, Ward A, Moller J. Modelling the health benefits and economic implications of implanting dual-chamber vs. single-chamber ventricular pacemakers in the UK. Europace. 06/ 2006;8(6):449-455.

66. Carter R, Hailey D. Economic evaluation of the cochlear implant. Int J Technol Assess Health Care. / 1999;15(3):520-530.

67. Cavaliere CM, Chung KC. A cost-utility analysis of nonsurgical management, total wrist arthroplasty, and total wrist arthrodesis in rheumatoid arthritis. J Hand Surg Am. 03/ 2010;35(3):379-391.

68. Chang JS, Smiddy WE. Cost-effectiveness of retinal detachment repair. Ophthalmology. 04/ 2014;121(4):946-951.

69. Chang JS, Smiddy WE. Cost evaluation of surgical and pharmaceutical options in treatment for vitreomacular adhesions and macular holes. Ophthalmology. 09/ 2014;121(9):1720-1726.

70. Chang RW, Pellisier JM, Hazen GB. A cost-effectiveness analysis of total hip arthroplasty for osteoarthritis of the hip. JAMA. 03/20 1996;275(11):858-865.

71. Chang SL, Cipriano LE, Harshman LC, Garber AM, Chung BI. Cost-effectiveness analysis of nephron sparing options for the management of small renal masses. J Urol. 05/ 2011;185(5):1591-1597.

72. Chen AT, Pedtke A, Kobs JK, Edwards GS, Coughlin RR, Gosselin RA. Volunteer orthopedic surgical trips in Nicaragua: a cost-effectiveness evaluation. World J Surg. 12/ 2012;36(12):2802-2808.

73. Chen JM, Amoodi H, Mittmann N. Cost-utility analysis of bilateral cochlear implantation in adults: a health economic assessment from the perspective of a publicly funded program. Laryngoscope. 06/ 2014;124(6):1452-1458.

74. Chen NC, Shauver MJ, Chung KC. Cost-effectiveness of open partial fasciectomy, needle aponeurotomy, and collagenase injection for dupuytren contracture. J Hand Surg Am. 11/ 2011;36(11):1826-1834.

75. Cheng CH, Sanders GD, Hlatky MA, et al. Cost-effectiveness of radiofrequency ablation for supraventricular tachycardia. Ann Intern Med. 12/05 2000;133(11):864-876.

76. Chew C, O'Dwyer PJ, Edwards R. Health service cost associated with percutaneous vertebroplasty in patients with spinal metastases. Clin Radiol. 08/ 2013;68(8):776-779.

77. Chongqing T, Liubao P, Xiaohui Z, et al. Cost-utility analysis of the newly recommended adjuvant chemotherapy for resectable gastric cancer patients in the 2011 Chinese National Comprehensive Cancer Network (NCCN) Clinical Practice Guidelines in Oncology: Gastric Cancer. Pharmacoeconomics. 03/ 2014;32(3):235-243.

78. Chuck AW, Hailey D, Jacobs P, Perry DC. Cost-effectiveness and budget impact of adjunctive hyperbaric oxygen therapy for diabetic foot ulcers. Int J Technol Assess Health Care. / 2008;24(2):178-183.

79. Chung KC, Oda T, Saddawi-Konefka D, Shauver MJ. An economic analysis of hand transplantation in the United States. Plast Reconstr Surg. 02/ 2010;125(2):589-598.

80. Chung KC, Walters MR, Greenfield ML, Chernew ME. Endoscopic versus open carpal tunnel release: a cost-effectiveness analysis. Plast Reconstr Surg. 09/ 1998;102(4):1089-1099.

81. Church J, Goodall S, Norman R, Haas M. The cost-effectiveness of falls prevention interventions for older community-dwelling Australians. Aust N Z J Public Health. 06/ 2012;36(3):241-248.

82. Clarke A, Pulikottil-Jacob R, Connock M, et al. Cost-effectiveness of left ventricular assist devices (LVADs) for patients with advanced heart failure: analysis of the British NHS bridge to transplant (BTT) program. Int J Cardiol. 02/15 2014;171(3):338-345.

83. Clement ND, MacDonald D, Gaston P. Hip arthroscopy for femoroacetabular impingement: a health economic analysis. Hip Int. / 2014;24(5):457-464.

84. Clermont G, Kong L, Weissfeld LA, et al. The effect of pulmonary artery catheter use on costs and long-term outcomes of acute lung injury. PLoS One. / 2011;6(7).

85. Close A, Robertson C, Rushton S, et al. Comparative cost-effectiveness of robot-assisted and standard laparoscopic prostatectomy as alternatives to open radical prostatectomy for treatment of men with localised prostate cancer: a health technology assessment from the perspective of the UK National Health Service. Eur Urol. 09/ 2013;64(3):361-369.

86. Coast J. Reprocessing data to form QALYs. BMJ. 07/11 1992;305(6845):87-90.

87. Cohen DJ, Bakhai A, Shi C, et al. Cost-effectiveness of sirolimus-eluting stents for treatment of complex coronary stenoses: results from the Sirolimus-Eluting Balloon Expandable Stent in the Treatment of Patients With De Novo Native Coronary Artery Lesions (SIRIUS) trial. Circulation. 08/03 2004;110(5):508-514.

88. Cohen DJ, Breall JA, Ho KK, et al. Evaluating the potential cost-effectiveness of stenting as a treatment for symptomatic single-vessel coronary disease. Use of a decision-analytic model. Circulation. 04/ 1994;89(4):1859-1874.

89. Cohen DJ, Sukin CA. Cost-effectiveness of coronary interventions. Heart. 10/ 1997;78 Suppl 2:7-10.

90. Cohen DJ, Taira DA, Berezin R, et al. Cost-effectiveness of coronary stenting in acute myocardial infarction: results from the stent primary angioplasty in myocardial infarction (stent-PAMI) trial. Circulation. 12/18 2001;104(25):3039-3045.

91. Comay D, Adam V, da Silveira EB, Kennedy W, Mayrand S, Barkun AN. The Stretta procedure versus proton pump inhibitors and laparoscopic Nissen fundoplication in the management of gastroesophageal reflux disease: a cost-effectiveness analysis. Can J Gastroenterol. 06/ 2008;22(6):552-558.

92. Comay D, Blackhouse G, Goeree R, Armstrong D, Marshall JK. Photodynamic therapy for Barrett's esophagus with high-grade dysplasia: a cost-effectiveness analysis. Can J Gastroenterol. 04/ 2007;21(4):217-222.

93. Cooperberg MR, Ramakrishna NR, Duff SB, et al. Primary treatments for clinically localised prostate cancer: a comprehensive lifetime cost-utility analysis. BJU Int. 03/ 2013;111(3):437-450.

94. Corlew DS. Estimation of impact of surgical disease through economic modeling of cleft lip and palate care. World J Surg. 03/ 2010;34(3):391-396.

95. Craig BM, Tseng DS. Cost-effectiveness of gastric bypass for severe obesity. Am J Med. 10/15 2002;113(6):491-498.

96. Cucchetti A, Piscaglia F, Cescon M, et al. Cost-effectiveness of hepatic resection versus percutaneous radiofrequency ablation for early hepatocellular carcinoma. J Hepatol. 08/ 2013;59(2):300-307.

97. Culligan PJ, Myers JA, Goldberg RP, Blackwell L, Gohmann SF, Abell TD. Elective cesarean section to prevent anal incontinence and brachial plexus injuries associated with macrosomia--a decision analysis. Int Urogynecol J Pelvic Floor Dysfunct. / 2005;16(1):19-28.

98. Cummins JS, Tomek IM, Kantor SR, Furnes O, Engesaeter LB, Finlayson SRG. Cost-effectiveness of antibiotic-impregnated bone cement used in primary total hip arthroplasty. J Bone Joint Surg Am. 03/01 2009;91(3):634-641.

99. Cunningham SJ, Sculpher M, Sassi F, Manca A. A cost-utility analysis of patients undergoing orthognathic treatment for the management of dentofacial disharmony. Br J Oral Maxillofac Surg. 02/ 2003;41(1):32-35.

100. Cuthbertson BH, Campbell MK, Stott SA, et al. A pragmatic multi-centre randomised controlled trial of fluid loading in high-risk surgical patients undergoing major elective surgery--the FOCCUS study. Crit Care. / 2011;15(6).

101. Dageforde LA, Landman MP, Feurer ID, Poulose B, Pinson CW, Moore DE. A cost-effectiveness analysis of early vs late reconstruction of iatrogenic bile duct injuries. J Am Coll Surg. 06/ 2012;214(6):919-927.

102. Dams J, Siebert U, Bornschein B, et al. Cost-effectiveness of deep brain stimulation in patients with Parkinson's disease. Mov Disord. 06/ 2013;28(6):763-771.

103. Davis MC, Than KD, Garton HJ. Cost effectiveness of a short-term pediatric neurosurgical brigade to guatemala. World Neurosurg. 12/ 2014;82(6):974-979.

104. de Verteuil RM, Hernandez RA, Vale L. Economic evaluation of laparoscopic surgery for colorectal cancer. Int J Technol Assess Health Care. / 2007;23(4):464-472.

105. de Vries SO, Visser K, de Vries JA, Wong JB, Donaldson MC, Hunink MGM. Intermittent claudication: cost-effectiveness of revascularization versus exercise therapy. Radiology. 01/ 2002;222(1):25-36.

106. de Wit GA, Ramsteijn PG, de Charro FT. Economic evaluation of end stage renal disease treatment. Health Policy. 06/ 1998;44(3):215-232.

107. DiSantostefano RL, Biddle AK, Lavelle JP. The long-term cost effectiveness of treatments for benign prostatic hyperplasia. Pharmacoeconomics. / 2006;24(2):171-191.

108. Disselhoff BCVM, Buskens E, Kelder JC, der Kinderen DJ, Moll FL. Randomised comparison of costs and cost-effectiveness of cryostripping and endovenous laser ablation for varicose veins: 2-year results. Eur J Vasc Endovasc Surg. 03/ 2009;37(3):357-363.

109. Diwakar L, Morris RK, Barton P, Middleton LJ, Kilby MD, Roberts TE. Evaluation of the cost effectiveness of vesico-amniotic shunting in the management of congenital lower urinary tract obstruction (based on data from the PLUTO Trial). PLoS One. / 2013;8(12).

110. Doble B, Blackhouse G, Goeree R, Xie F. Cost-effectiveness of the Edwards SAPIEN transcatheter heart valve compared with standard management and surgical aortic valve replacement in patients with severe symptomatic aortic stenosis: a Canadian perspective. J Thorac Cardiovasc Surg. 07/ 2013;146(1):52-60.

111. Dominguez J, Harrison R, Atal R. Cost-benefit estimation of cadaveric kidney transplantation: the case of a developing country. Transplant Proc. / 2011;43(6):2300-2304.

112. Dong D, Tan A, Mehta JS, Tan D, Finkelstein EA. Cost-effectiveness of osteo-odonto keratoprosthesis in Singapore. Am J Ophthalmol. 01/ 2014;157(1):78-84.

113. Dorenkamp M, Bonaventura K, Leber AW, et al. Potential lifetime cost-effectiveness of catheter-based renal sympathetic denervation in patients with resistant hypertension. Eur Heart J. 02/ 2013;34(6):451-461.

114. Duwelius PJ, Brenner JS, Reyner DP, George JC. Cost Effectiveness of Minimally Invasive Total Hip Arthroplasty. Seminars in Arthroplasty. 2008;19(2):186-193.

115. Edlin R, Tubeuf S, Achten J, Parsons N, Costa M. Cost-effectiveness of total hip arthroplasty versus resurfacing arthroplasty: economic evaluation alongside a clinical trial. BMJ Open. / 2012;2(5).

116. Eefting F, Nathoe H, van Dijk D, et al. Randomized comparison between stenting and off-pump bypass surgery in patients referred for angioplasty. Circulation. 12/09 2003;108(23):2870-2876.

117. Eeson G, Birabwa-Male D, Pennington M, Blair GK. Costs and cost-effectiveness of pediatric inguinal hernia repair in Uganda. World J Surg. 02/ 2015;39(2):343-349.

118. Egle JP, McKendrick A, Mittal VK, Sosa F. Short-term surgical mission to the Dominican Republic: a cost-benefit analysis. Int J Surg. 10/ 2014;12(10):1045-1049.

119. Eisenstein EL, Leon MB, Kandzari DE, et al. Long-term clinical and economic analysis of the Endeavor zotarolimus-eluting stent versus the cypher sirolimus-eluting stent: 3-year results from the ENDEAVOR III trial (Randomized Controlled Trial of the Medtronic Endeavor Drug [ABT-578] Eluting Coronary Stent System Versus the Cypher Sirolimus-Eluting Coronary Stent System in De Novo Native Coronary Artery Lesions). JACC Cardiovasc Interv. 12/ 2009;2(12):1199-1207.

120. Ekman M, Sjogren I, James S. Cost-effectiveness of the Taxus paclitaxel-eluting stent in the Swedish healthcare system. Scand Cardiovasc J. 02/ 2006;40(1):17-24.

121. Epstein D, Sculpher MJ, Powell JT, Thompson SG, Brown LC, Greenhalgh RM. Long-term cost-effectiveness analysis of endovascular versus open repair for abdominal aortic aneurysm based on four randomized clinical trials. Br J Surg. 05/ 2014;101(6):623-631.

122. Erstad BL. Cost-effectiveness of proton pump inhibitor therapy for acute peptic ulcer-related bleeding. Crit Care Med. 06/ 2004;32(6):1277-1283.

123. Eskelinen E, Rasanen P, Alback A, et al. Effectiveness of superficial venous surgery in terms of quality-adjusted life years and costs. Scand J Surg. / 2009;98(4):229-233.

124. Esnaola NF, Lazarides SN, Mentzer SJ, Kuntz KM. Outcomes and cost-effectiveness of alternative staging strategies for non-small-cell lung cancer. J Clin Oncol. 01/01 2002;20(1):263-273.

125. Fairbairn TA, Meads DM, Hulme C, et al. The cost-effectiveness of transcatheter aortic valve implantation versus surgical aortic valve replacement in patients with severe aortic stenosis at high operative risk. Heart. 07/ 2013;99(13):914-920.

126. Faria GR, Preto JR, Costa-Maia J. Gastric bypass is a cost-saving procedure: results from a comprehensive Markov model. Obes Surg. 04/ 2013;23(4):460-466.

127. Farshad M, Gerber C, Meyer DC, Schwab A, Blank PR, Szucs T. Reconstruction versus conservative treatment after rupture of the anterior cruciate ligament: cost effectiveness analysis. BMC Health Serv Res. / 2011;11:317-317.

128. Faucett SC, Collinge CA, Koval KJ. Is reconstruction nailing of all femoral shaft fractures cost effective? A decision analysis. J Orthop Trauma. 11/ 2012;26(11):624-632.

129. Faucett SC, Genuario JW, Tosteson ANA, Koval KJ. Is prophylactic fixation a cost-effective method to prevent a future contralateral fragility hip fracture? J Orthop Trauma. 02/ 2010;24(2):65-74.

130. Fawsitt CG, Bourke J, Greene RA, Everard CM, Murphy A, Lutomski JE. At what price? A cost-effectiveness analysis comparing trial of labour after previous caesarean versus elective repeat caesarean delivery. PLoS One. / 2013;8(3).

131. Fearon WF, Shilane D, Pijls NHJ, et al. Cost-effectiveness of percutaneous coronary intervention in patients with stable coronary artery disease and abnormal fractional flow reserve. Circulation. 09/17 2013;128(12):1335-1340.

132. Fehlings MG, Jha NK, Hewson SM, Massicotte EM, Kopjar B, Kalsi-Ryan S. Is surgery for cervical spondylotic myelopathy cost-effective? A cost-utility analysis based on data from the AOSpine North America prospective CSM study. J Neurosurg Spine. 09/ 2012;17(1 Suppl):89-93.

133. Fernandez-Fairen M, Murcia A, Torres A, Hernandez-Vaquero D, Menzie AM. Is anterior cervical fusion with a porous tantalum implant a cost-effective method to treat cervical disc disease with radiculopathy? Spine (Phila Pa 1976). 09/15 2012;37(20):1734-1741.

134. Fischer CR, Terran J, Lonner B, et al. Factors predicting cost-effectiveness of adult spinal deformity surgery at 2 years. Spine Deformity. 2014;2(5):415-422.

135. Fitzsimmons D, Phillips CJ, Bennett H, et al. Cost-effectiveness of different strategies to manage patients with sciatica. Pain. 07/ 2014;155(7):1318-1327.

136. Fjalestad T, Hole MO, Jorgensen JJ, Stromsoe K, Kristiansen IS. Health and cost consequences of surgical versus conservative treatment for a comminuted proximal humeral fracture in elderly patients. Injury. 06/ 2010;41(6):599-605.

137. Forbes JF, Adam DJ, Bell J, et al. Bypass versus Angioplasty in Severe Ischaemia of the Leg (BASIL) trial: Health-related quality of life outcomes, resource utilization, and cost-effectiveness analysis. J Vasc Surg. 05/ 2010;51(5 Suppl):51.

138. Frick KD, Clark MA, Steinwachs DM, et al. Financial and quality-of-life burden of dysfunctional uterine bleeding among women agreeing to obtain surgical treatment. Womens Health Issues. / 2009;19(1):70-78.

139. Fritzell P, Berg S, Borgstrom F, Tullberg T, Tropp H. Cost effectiveness of disc prosthesis versus lumbar fusion in patients with chronic low back pain: randomized controlled trial with 2-year follow-up. Eur Spine J. 07/ 2011;20(7):1001-1011.

140. Fritzell P, Ohlin A, Borgstrom F. Cost-effectiveness of balloon kyphoplasty versus standard medical treatment in patients with osteoporotic vertebral compression fracture: a Swedish multicenter randomized controlled trial with 2-year follow-up. Spine (Phila Pa 1976). 12/15 2011;36(26):2243-2251.

141. Fujiike K, Mizuno Y, Hiratsuka Y, Yamada M. Quality of life and cost-utility assessment after strabismus surgery in adults. Jpn J Ophthalmol. 05/ 2011;55(3):268-276.

142. Furlan JC, Chan KKW, Sandoval GA, et al. The combined use of surgery and radiotherapy to treat patients with epidural cord compression due to metastatic disease: a cost-utility analysis. Neuro Oncol. 05/ 2012;14(5):631-640.

143. Gaetani P, Rodriguez y Baena R, Klersy C, Adinolfi D, Infuso L. A cost-effectiveness analysis on different surgical strategies for intracranial aneurysms. J Neurosurg Sci. 06/ 1998;42(2):69-78.

144. Gandjour A, Weyler E-J. Cost-effectiveness of referrals to high-volume hospitals: an analysis based on a probabilistic Markov model for hip fracture surgeries. Health Care Manag Sci. 11/ 2006;9(4):359-369.

145. Garry R, Fountain J, Brown J, et al. EVALUATE hysterectomy trial: a multicentre randomised trial comparing abdominal, vaginal and laparoscopic methods of hysterectomy. Health Technol Assess. 06/ 2004;8(26):1-154.

146. Gazelle GS, Hunink MGM, Kuntz KM, et al. Cost-effectiveness of hepatic metastasectomy in patients with metastatic colorectal carcinoma: a state-transition Monte Carlo decision analysis. Ann Surg. 04/ 2003;237(4):544-555.

147. Gazelle GS, McMahon PM, Beinfeld MT, Halpern EF, Weinstein MC. Metastatic colorectal carcinoma: cost-effectiveness of percutaneous radiofrequency ablation versus that of hepatic resection. Radiology. 12/ 2004;233(3):729-739.

148. Gelsomino S, Lorusso R, Livi U, et al. Cost and cost-effectiveness of cardiac surgery in elderly patients. J Thorac Cardiovasc Surg. 11/ 2011;142(5):1062-1073.

149. Gerlier L, Lamotte M, Wille M, et al. The cost utility of autologous chondrocytes implantation using ChondroCelect® in symptomatic knee cartilage lesions in Belgium. Pharmacoeconomics. / 2010;28(12):1129-1146.

150. Giannicola G, Bullitta G, Sacchetti FM, et al. Change in quality of life and cost/utility analysis in open stage-related surgical treatment of elbow stiffness. Orthopedics. 07/ 2013;36(7):923-930.

151. Gilbert SA, Grobman WA, Landon MB, et al. Cost-effectiveness of trial of labor after previous cesarean in a minimally biased cohort. Am J Perinatol. 01/ 2013;30(1):11-20.

152. Gilbert SA, Grobman WA, Landon MB, et al. Lifetime cost-effectiveness of trial of labor after cesarean in the United States. Value Health. / 2013;16(6):953-964.

153. Ginsberg G, Adunsky A, Rasooly I. A cost-utility analysis of a comprehensive orthogeriatric care for hip fracture patients, compared with standard of care treatment. Hip Int. / 2013;23(6):570-575.

154. Giorgio Calori M, Capanna R, Colombo M, et al. Cost effectiveness of tibial nonunion treatment: A comparison between rhBMP-7 and autologous bone graft in two Italian centres. Injury. 12/ 2013;44(12):1871-1879.

155. Givon U, Ginsberg GM, Horoszowski H, Shemer J. Cost-utility analysis of total hip arthroplasties. Technology assessment of surgical procedures by mailed questionnaires. Int J Technol Assess Health Care. / 1998;14(4):735-742.

156. Glassman SD, Polly DW, Dimar JR, Carreon LY. The cost effectiveness of single-level instrumented posterolateral lumbar fusion at 5 years after surgery. Spine (Phila Pa 1976). 04/20 2012;37(9):769-774.

157. Goeree R, Hopkins R, Marshall JK, et al. Cost-utility of laparoscopic Nissen fundoplication versus proton pump inhibitors for chronic and controlled gastroesophageal reflux disease: a 3-year prospective randomized controlled trial and economic evaluation. Value Health. / 2011;14(2):263-273.

158. Gold HT, Hayes MK. Cost effectiveness of new breast cancer radiotherapy technologies in diverse populations. Breast Cancer Res Treat. 11/ 2012;136(1):221-229.

159. Gordon LG, Hirst NG, Mayne GC, et al. Modeling the cost-effectiveness of strategies for treating esophageal adenocarcinoma and high-grade dysplasia. J Gastrointest Surg. 08/ 2012;16(8):1451-1461.

160. Gosselin RA, Gialamas G, Atkin DM. Comparing the cost-effectiveness of short orthopedic missions in elective and relief situations in developing countries. World J Surg. 05/ 2011;35(5):951-955.

161. Gosselin RA, Heitto M. Cost-effectiveness of a district trauma hospital in Battambang, Cambodia. World J Surg. 11/ 2008;32(11):2450-2453.

162. Gosselin RA, Maldonado A, Elder G. Comparative cost-effectiveness analysis of two MSF surgical trauma centers. World J Surg. 03/ 2010;34(3):415-419.

163. Gosselin RA, Thind A, Bellardinelli A. Cost/DALY averted in a small hospital in Sierra Leone: what is the relative contribution of different services? World J Surg. 04/ 2006;30(4):505-511.

164. Gothesen O, Slover J, Havelin L, Askildsen JE, Malchau H, Furnes O. An economic model to evaluate cost-effectiveness of computer assisted knee replacement surgery in Norway. BMC Musculoskelet Disord. / 2013;14:202-202.

165. Govers TM, Takes RP, Baris Karakullukcu M, et al. Management of the N0 neck in early stage oral squamous cell cancer: a modeling study of the cost-effectiveness. Oral Oncol. 08/ 2013;49(8):771-777.

166. Grant AM, Boachie C, Cotton SC, et al. Clinical and economic evaluation of laparoscopic surgery compared with medical management for gastro-oesophageal reflux disease: 5-year follow-up of multicentre randomised trial (the REFLUX trial). Health Technol Assess. 06/ 2013;17(22):1-167.

167. Greenblatt WH, Hur C, Knudsen AB, Evans JA, Chung DC, Gazelle GS. Cost-effectiveness of prophylactic surgery for duodenal cancer in familial adenomatous polyposis. Cancer Epidemiol Biomarkers Prev. 10/ 2009;18(10):2677-2684.

168. Greenhalgh J, Bagust A, Boland A, et al. Prasugrel for the treatment of acute coronary artery syndromes with percutaneous coronary intervention. Health Technol Assess. 05/ 2010;14 Suppl 1:31-38.

169. Gregor JC, Ponich TP, Detsky AS. Should ERCP be routine after an episode of "idiopathic" pancreatitis? A cost-utility analysis. Gastrointest Endosc. 08/ 1996;44(2):118-123.

170. Greiner W, Obermann K, Schulenburg JM. Socio-economic evaluation of kidney transplantation in Germany. Archives of Hellenic Medicine. 2001;18(2):147-155.

171. Greving JP, Vernooij F, Heintz APM, van der Graaf Y, Buskens E. Is centralization of ovarian cancer care warranted? A cost-effectiveness analysis. Gynecol Oncol. 04/ 2009;113(1):68-74.

172. Griffiths UK, Bozzani FM, Gheorghe A, Mwenge L, Gilbert C. Cost-effectiveness of eye care services in Zambia. Cost Eff Resour Alloc. / 2014;12(1):6-6.

173. Groen H, Moers C, Smits JM, et al. Cost-effectiveness of hypothermic machine preservation versus static cold storage in renal transplantation. Am J Transplant. 07/ 2012;12(7):1824-1830.

174. Groen H, van der Bij W, Koeter GH, TenVergert EM. Cost-effectiveness of lung transplantation in relation to type of end-stage pulmonary disease. Am J Transplant. 07/ 2004;4(7):1155-1162.

175. Gupta OP, Brown GC, Brown MM. A value-based medicine cost-utility analysis of idiopathic epiretinal membrane surgery. Am J Ophthalmol. 05/ 2008;145(5):923-928.

176. Gurusamy K, Wilson E, Burroughs AK, Davidson BR. Intra-operative vs pre-operative endoscopic sphincterotomy in patients with gallbladder and common bile duct stones: cost-utility and value-of-information analysis. Appl Health Econ Health Policy. 01/01 2012;10(1):15-29.

177. Hak A, Li CS, Bhandari M. Cost-effectiveness and economic impact of the KineSpring® Knee Implant System in the treatment of knee osteoarthritis in the United Kingdom. J Long Term Eff Med Implants. / 2013;23(2-3):199-210.

178. Hamidi V, Andersen MH, Oyen O, Mathisen L, Fosse E, Kristiansen IS. Cost effectiveness of open versus laparoscopic living-donor nephrectomy. Transplantation. 03/27 2009;87(6):831-838.

179. Hancock-Howard RL, Feindel CM, Rodes-Cabau J, Webb JG, Thompson AK, Banz K. Cost effectiveness of transcatheter aortic valve replacement compared to medical management in inoperable patients with severe aortic stenosis: Canadian analysis based on the PARTNER Trial Cohort B findings. J Med Econ. / 2013;16(4):566-574.

180. Hansen KS, Chapman G. Setting priorities for the health care sector in Zimbabwe using cost-effectiveness analysis and estimates of the burden of disease. Cost Eff Resour Alloc. / 2008;6:14-14.

181. Hansson E, Hansson T. The cost-utility of lumbar disc herniation surgery. Eur Spine J. 03/ 2007;16(3):329-337.

182. Hatoum HT, Fierlinger AL, Lin S-J, Altman RD. Cost-effectiveness analysis of intra-articular injections of a high molecular weight bioengineered hyaluronic acid for the treatment of osteoarthritis knee pain. J Med Econ. 05/ 2014;17(5):326-337.

183. Hattori N, Hirayama T, Katayama Y. Cost-effectiveness analysis of intrathecal baclofen therapy in Japan. Neurol Med Chir (Tokyo). / 2012;52(7):482-487.

184. Hayashi K, Abe K, Yano F, Watanabe S, Iwasaki Y, Kosuda S. Should mediastinoscopy actually be incorporated into the FDG PET strategy for patients with non-small cell lung carcinoma? Ann Nucl Med. 07/ 2005;19(5):393-398.

185. Hayes JH, Ollendorf DA, Pearson SD, et al. Observation versus initial treatment for men with localized, low-risk prostate cancer: a cost-effectiveness analysis. Ann Intern Med. 06/18 2013;158(12):853-860.

186. Hayes JL, Hansen P. Is laparoscopic colectomy for cancer cost-effective relative to open colectomy? ANZ J Surg. 09/ 2007;77(9):782-786.

187. Heintzbergen S, Kulin NA, Ijzerman MJ, et al. Cost-utility of metal-on-metal hip resurfacing compared to conventional total hip replacement in young active patients with osteoarthritis. Value Health. / 2013;16(6):942-952.

188. Henriksson M, Lundgren F, Carlsson P. Cost-effectiveness of endarterectomy in patients with asymptomatic carotid artery stenosis. Br J Surg. 06/ 2008;95(6):714-720.

189. Higashi H, Barendregt JJ. Cost-effectiveness of total hip and knee replacements for the Australian population with osteoarthritis: discrete-event simulation model. PLoS One. / 2011;6(9).

190. Hiratsuka Y, Yamada M, Akune Y, et al. Cost-utility analysis of cataract surgery in Japan: a probabilistic Markov modeling study. Jpn J Ophthalmol. 07/ 2013;57(4):391-401.

191. Hiratsuka Y, Yamada M, Murakami A, et al. Cost-effectiveness of cataract surgery in Japan. Jpn J Ophthalmol. 07/ 2011;55(4):333-342.

192. Hirneiss C, Neubauer AS, Niedermeier A, Messmer EM, Ulbig M, Kampik A. Cost utility for penetrating keratoplasty in patients with poor binocular vision. Ophthalmology. 12/ 2006;113(12):2176-2180.

193. Hisashige A, Yoshida S, Kodaira S. Cost-effectiveness of adjuvant chemotherapy with uracil-tegafur for curatively resected stage III rectal cancer. Br J Cancer. 10/21 2008;99(8):1232-1238.

194. Hjelmgren J, Ghatnekar O, Reimer J, et al. Estimating the value of novel interventions for Parkinson's disease: an early decision-making model with application to dopamine cell replacement. Parkinsonism Relat Disord. 10/ 2006;12(7):443-452.

195. Ho KM, Honeybul S, Lind CRP, Gillett GR, Litton E. Cost-effectiveness of decompressive craniectomy as a lifesaving rescue procedure for patients with severe traumatic brain injury. J Trauma. 12/ 2011;71(6):1637-1644.

196. Hoerger TJ, Zhang P, Segel JE, Kahn HS, Barker LE, Couper S. Cost-effectiveness of bariatric surgery for severely obese adults with diabetes. Diabetes Care. 09/ 2010;33(9):1933-1939.

197. Hofmeijer J, van der Worp HB, Kappelle LJ, Eshuis S, Algra A, Greving JP. Cost-effectiveness of surgical decompression for space-occupying hemispheric infarction. Stroke. 10/ 2013;44(10):2923-2925.

198. Hogendoorn W, Hunink MGM, Schlosser FJV, Moll FL, Muhs BE, Sumpio BE. A comparison of open and endovascular revascularization for chronic mesenteric ischemia in a clinical decision model. J Vasc Surg. 09/ 2014;60(3):715-725.

199. Hogendoorn W, Schlosser FJV, Moll FL, Muhs BE, Hunink MGM, Sumpio BE. Decision analysis model of open repair versus endovascular treatment in patients with asymptomatic popliteal artery aneurysms. J Vasc Surg. 03/ 2014;59(3):651-662.

200. Hogenhuis W, Stevens SK, Wang P, et al. Cost-effectiveness of radiofrequency ablation compared with other strategies in Wolff-Parkinson-White syndrome. Circulation. 11/ 1993;88(5 Pt 2):437-446.

201. Hohwu L, Borre M, Ehlers L, Venborg Pedersen K. A short-term cost-effectiveness study comparing robot-assisted laparoscopic and open retropubic radical prostatectomy. J Med Econ. / 2011;14(4):403-409.

202. Holler D, Claes C, Von Der Schulenburg JMG. Cost-utility analysis of treating severe peripheral arterial occlusive disease. International Journal of Angiology. 2006;15(1):25-33.

203. Hornberger JC, Best JH, Garrison LP. Cost-effectiveness of repeat medical procedures: kidney transplantation as an example. Med Decis Making. / 1997;17(4):363-372.

204. Houlind K, Kjeldsen BJ, Madsen SN, et al. OPCAB surgery is cost-effective for elderly patients. Scand Cardiovasc J. 06/ 2013;47(3):185-192.

205. Howard K, Lord SJ, Speer A, Gibson RN, Padbury R, Kearney B. Value of magnetic resonance cholangiopancreatography in the diagnosis of biliary abnormalities in postcholecystectomy patients: a probabilistic cost-effectiveness analysis of diagnostic strategies. Int J Technol Assess Health Care. / 2006;22(1):109-118.

206. Howard K, Salkeld G, White S, et al. The cost-effectiveness of increasing kidney transplantation and home-based dialysis. Nephrology (Carlton). 02/ 2009;14(1):123-132.

207. Hu D, Grossman D, Levin C, Blanchard K, Goldie SJ. Cost-effectiveness analysis of alternative first-trimester pregnancy termination strategies in Mexico City. BJOG. 05/ 2009;116(6):768-779.

208. Huang ES, Gazelle GS, Hur C. Consensus guidelines in the management of branch duct intraductal papillary mucinous neoplasm: a cost-effectiveness analysis. Dig Dis Sci. 03/ 2010;55(3):852-860.

209. Hughes CD, Babigian A, McCormack S, et al. The clinical and economic impact of a sustained program in global plastic surgery: valuing cleft care in resource-poor settings. Plast Reconstr Surg. 07/ 2012;130(1):94.

210. Hulscher JBF, van Sandick JW, de Boer AGEM, et al. Extended transthoracic resection compared with limited transhiatal resection for adenocarcinoma of the esophagus. N Engl J Med. 11/21 2002;347(21):1662-1669.

211. Hunink MG, Bult JR, de Vries J, Weinstein MC. Uncertainty in decision models analyzing cost-effectiveness: the joint distribution of incremental costs and effectiveness evaluated with a nonparametric bootstrap method. Med Decis Making. / 1998;18(3):337-346.

212. Hunter RM, Isaac M, Frigiola A, Blundell D, Brown K, Bull K. Lifetime costs and outcomes of repair of Tetralogy of Fallot compared to natural progression of the disease: Great Ormond Street Hospital cohort. PLoS One. / 2013;8(3).

213. Hur C, Choi SE, Rubenstein JH, et al. The cost effectiveness of radiofrequency ablation for Barrett's esophagus. Gastroenterology. 09/ 2012;143(3):567-575.

214. Ikramuddin S, Klingman D, Swan T, Minshall ME. Cost-effectiveness of Roux-en-Y gastric bypass in type 2 diabetes patients. Am J Manag Care. 09/ 2009;15(9):607-615.

215. Ilbawi AM, Einterz EM, Nkusu D. Obstacles to surgical services in a rural Cameroonian district hospital. World J Surg. 06/ 2013;37(6):1208-1215.

216. Imperiale TF, Klein RW, Chalasani N. Cost-effectiveness analysis of variceal ligation vs. beta-blockers for primary prevention of variceal bleeding. Hepatology. 04/ 2007;45(4):870-878.

217. In H, Pearce EN, Wong AK, Burgess JF, McAneny DB, Rosen JE. Treatment options for Graves disease: a cost-effectiveness analysis. J Am Coll Surg. 08/ 2009;209(2):170-179.

218. Indinnimeo M, Ratto C, Moschella CM, Fiore A, Brosa M, Giardina S. Sacral neuromodulation for the treatment of fecal incontinence: analysis of cost-effectiveness. Dis Colon Rectum. 12/ 2010;53(12):1661-1669.

219. Ishida K, Imai H, Ogasawara K, et al. Cost-utility of living donor liver transplantation in a single Japanese center. Hepatogastroenterology. / 2006;53(70):588-591.

220. Iva G. Cost-effectiveness analysis of percutaneous coronary intervention versus thrombolytic therapy in patients with an ST-elevated myocardial infarction. Serbian Journal of Experimental and Clinical Research. 2011;12(4):147-152.

221. Jacklin P, Duckett J. A decision-analytic Markov model to compare the cost-utility of anterior repair augmented with synthetic mesh compared with non-mesh repair in women with surgically treated prolapse. BJOG. 01/ 2013;120(2):217-223.

222. Jaisson-Hot I, Flourie B, Descos L, Colin C. Management for severe Crohn's disease: a lifetime cost-utility analysis. Int J Technol Assess Health Care. / 2004;20(3):274-279.

223. Jassal SV, Krahn MD, Naglie G, et al. Kidney transplantation in the elderly: a decision analysis. J Am Soc Nephrol. 01/ 2003;14(1):187-196.

224. Javitt J, Dei Cas R, Chiang YP. Cost-effectiveness of screening and cryotherapy for threshold retinopathy of prematurity. Pediatrics. 05/ 1993;91(5):859-866.

225. Jay CL, Skaro AI, Ladner DP, et al. Comparative effectiveness of donation after cardiac death versus donation after brain death liver transplantation: Recognizing who can benefit. Liver Transpl. 06/ 2012;18(6):630-640.

226. Jensen CE, Sorensen P, Petersen KD. In Denmark kidney transplantation is more cost-effective than dialysis. Dan Med J. 03/ 2014;61(3).

227. Johner A, Raymakers A, Wiseman SM. Cost utility of early versus delayed laparoscopic cholecystectomy for acute cholecystitis. Surg Endosc. 01/ 2013;27(1):256-262.

228. Johnsen LG, Hellum C, Storheim K, et al. Cost-effectiveness of total disc replacement versus multidisciplinary rehabilitation in patients with chronic low back pain: a Norwegian multicenter RCT. Spine (Phila Pa 1976). 01/01 2014;39(1):23-32.

229. Johnston SC, Gress DR, Kahn JG. Which unruptured cerebral aneurysms should be treated? A cost-utility analysis. Neurology. 06/10 1999;52(9):1806-1815.

230. Jordan J, Dowson H, Gage H, Jackson D, Rockall T. Laparoscopic versus open colorectal resection for cancer and polyps: a cost-effectiveness study. Clinicoecon Outcomes Res. / 2014;6:415-422.

231. Kaiser C, Brunner-La Rocca HP, Buser PT, et al. Incremental cost-effectiveness of drug-eluting stents compared with a third-generation bare-metal stent in a real-world setting: randomised Basel Stent Kosten Effektivitats Trial (BASKET). Lancet. 09/10 2005;366(9489):921-929.

232. Kaminota M. Cost-effectiveness analysis of dialysis and kidney transplants in Japan. Keio J Med. 06/ 2001;50(2):100-108.

233. Kapma MR, Dijksman LM, Reimerink JJ, et al. Cost-effectiveness and cost-utility of endovascular versus open repair of ruptured abdominal aortic aneurysm in the Amsterdam Acute Aneurysm Trial. Br J Surg. 02/ 2014;101(3):208-215.

234. Karuna ST, Thirlby R, Biehl T, Veenstra D. Cost-effectiveness of laparoscopy versus laparotomy for initial surgical evaluation and treatment of potentially resectable hepatic colorectal metastases: a decision analysis. J Surg Oncol. 04/01 2008;97(5):396-403.

235. Kato H, Okunaka T, Tsuchida T, Shibuya H, Fujino S, Ogawa K. Analysis of the Cost-effectiveness of Photodynamic Therapy in Early Stage Lung Cancer. Diagn Ther Endosc. / 1999;6(1):9-16.

236. Keating CL, Dixon JB, Moodie ML, et al. Cost-effectiveness of surgically induced weight loss for the management of type 2 diabetes: modeled lifetime analysis. Diabetes Care. 04/ 2009;32(4):567-574.

237. Kensinger CD, Dageforde LA, Moore DE. Can donors with high donor risk indices be used cost-effectively in liver transplantation in US Transplant Centers? Transpl Int. 11/ 2013;26(11):1063-1069.

238. Khan AA, Chaudhry SA, Sivagnanam K, Hassan AE, Suri MFK, Qureshi AI. Cost-effectiveness of carotid artery stent placement versus endarterectomy in patients with carotid artery stenosis. J Neurosurg. 07/ 2012;117(1):89-93.

239. Kiberd BA. Should hepatitis C-infected kidneys be transplanted in the United States? Transplantation. 04/15 1994;57(7):1068-1072.

240. Kiberd BA, Larson T. Estimating the benefits of solitary pancreas transplantation in nonuremic patients with type 1 diabetes mellitus: a theoretical analysis. Transplantation. 10/15 2000;70(7):1121-1127.

241. Kim AS, Nguyen-Huynh M, Johnston SC. A cost-utility analysis of mechanical thrombectomy as an adjunct to intravenous tissue-type plasminogen activator for acute large-vessel ischemic stroke. Stroke. 07/ 2011;42(7):2013-2018.

242. Kim S, Mortaz Hedjri S, Coyte PC, Rampersaud YR. Cost-utility of lumbar decompression with or without fusion for patients with symptomatic degenerative lumbar spondylolisthesis. Spine J. 01/ 2012;12(1):44-54.

243. Klazen CAH, Lohle PNM, de Vries J, et al. Vertebroplasty versus conservative treatment in acute osteoporotic vertebral compression fractures (Vertos II): an open-label randomised trial. Lancet. 09/25 2010;376(9746):1085-1092.

244. Kobelt G, Lundstrom M, Stenevi U. Cost-effectiveness of cataract surgery. Method to assess cost-effectiveness using registry data. J Cataract Refract Surg. 10/ 2002;28(10):1742-1749.

245. Koerkamp BG, Spronk S, Stijnen T, Hunink MGM. Value of information analyses of economic randomized controlled trials: the treatment of intermittent claudication. Value Health. / 2010;13(2):242-250.

246. Koffijberg H, Buskens E, Rinkel GJE. Aneurysm occlusion in elderly patients with aneurysmal subarachnoid haemorrhage: a cost-utility analysis. J Neurol Neurosurg Psychiatry. 07/ 2011;82(7):718-727.

247. Kok NFM, Adang EMM, Hansson BME, et al. Cost effectiveness of laparoscopic versus mini-incision open donor nephrectomy: a randomized study. Transplantation. 06/27 2007;83(12):1582-1587.

248. Kong CY, Meng L, Omer ZB, et al. MRI-guided focused ultrasound surgery for uterine fibroid treatment: a cost-effectiveness analysis. AJR Am J Roentgenol. 08/ 2014;203(2):361-371.

249. Kontsevaia AV, Suvorova EI, Khudiakov MB. [Economic efficiency of renal denervation in patients with resistant hypertension: results of Markov modeling]. Kardiologiia. / 2014;54(1):41-47.

250. Koo TS, Finkelstein E, Tan D, Mehta JS. Incremental cost-utility analysis of deep anterior lamellar keratoplasty compared with penetrating keratoplasty for the treatment of keratoconus. Am J Ophthalmol. 07/ 2011;152(1):40-47.

251. Krashin JW, Edelman AB, Nichols MD, Allen AJ, Caughey AB, Rodriguez MI. Prohibiting consent: what are the costs of denying permanent contraception concurrent with abortion care? Am J Obstet Gynecol. 07/ 2014;211(1):1-76.

252. Krummenauer F, Wolf C, Gunther KP, Kirschner S. Clinical benefit and cost effectiveness of total knee arthroplasty in the older patient. Eur J Med Res. / 2009;14:76-84.

253. Kulkarni GS, Alibhai SMH, Finelli A, et al. Cost-effectiveness analysis of immediate radical cystectomy versus intravesical Bacillus Calmette-Guerin therapy for high-risk, high-grade (T1G3) bladder cancer. Cancer. 12/01 2009;115(23):5450-5459.

254. Kumar K, Rizvi S. Cost-effectiveness of spinal cord stimulation therapy in management of chronic pain. Pain Med. 11/ 2013;14(11):1631-1649.

255. Kwon JS, Carey MS, Goldie SJ, Kim JJ. Cost-effectiveness analysis of treatment strategies for Stage I and II endometrial cancer. J Obstet Gynaecol Can. 02/ 2007;29(2):131-139.

256. Labrie J, van der Graaf Y, Buskens E, Tiersma SESM, van der Vaart HCH. Protocol for Physiotherapy Or TVT Randomised Efficacy Trial (PORTRET): a multicentre randomised controlled trial to assess the cost-effectiveness of the tension free vaginal tape versus pelvic floor muscle training in women with symptomatic moderate to severe stress urinary incontinence. BMC Womens Health. / 2009;9:24-24.

257. Lamotte M, Annemans L, Bridgewater B, Kendall S, Siebert M. A health economic evaluation of concomitant surgical ablation for atrial fibrillation. Eur J Cardiothorac Surg. 11/ 2007;32(5):702-710.

258. Landman MP, Feurer ID, Pinson CW, Moore DE. Which is more cost-effective under the MELD system: primary liver transplantation, or salvage transplantation after hepatic resection or after loco-regional therapy for hepatocellular carcinoma within Milan criteria? HPB (Oxford). 11/ 2011;13(11):783-791.

259. Lansingh VC, Carter MJ. Use of Global Visual Acuity Data in a time trade-off approach to calculate the cost utility of cataract surgery. Arch Ophthalmol. 09/ 2009;127(9):1183-1193.

260. Lansingh VC, Carter MJ, Martens M. Global cost-effectiveness of cataract surgery. Ophthalmology. 09/ 2007;114(9):1670-1678.

261. Laudano MA, Seklehner S, Chughtai B, et al. Cost-effectiveness analysis of tension-free vaginal tape vs burch colposuspension for female stress urinary incontinence in the USA. BJU Int. 07/ 2013;112(2):151-158.

262. Launois R, Henry B, Marty JR, et al. Chemonucleolysis versus surgical discectomy for sciatica secondary to lumbar disc herniation. A cost and quality-of-life evaluation. Pharmacoeconomics. 11/ 1994;6(5):453-463.

263. Lavender M, Craig N, Kerr R, Howel D. Computer simulation to estimate the effectiveness of carotid endarterectomy. J Health Serv Res Policy. 01/ 1998;3(1):6-11.

264. Lederle FA, Stroupe KT. Cost-effectiveness at two years in the VA Open Versus Endovascular Repair Trial. Eur J Vasc Endovasc Surg. 12/ 2012;44(6):543-548.

265. Lee L, Saleem A, Landry T, Latimer E, Chaudhury P, Feldman LS. Cost effectiveness of mesh prophylaxis to prevent parastomal hernia in patients undergoing permanent colostomy for rectal cancer. J Am Coll Surg. 01/ 2014;218(1):82-91.

266. Lee L, Sudarshan M, Li C, et al. Cost-effectiveness of minimally invasive versus open esophagectomy for esophageal cancer. Ann Surg Oncol. 11/ 2013;20(12):3732-3739.

267. Lee SJ, Anasetti C, Kuntz KM, Patten J, Antin JH, Weeks JC. The costs and cost-effectiveness of unrelated donor bone marrow transplantation for chronic phase chronic myelogenous leukemia. Blood. 12/01 1998;92(11):4047-4052.

268. Lee YY, Veerman JL, Barendregt JJ. The cost-effectiveness of laparoscopic adjustable gastric banding in the morbidly obese adult population of Australia. PLoS One. / 2013;8(5).

269. Leon MB, Kandzari DE, Eisenstein EL, et al. Late safety, efficacy, and cost-effectiveness of a zotarolimus-eluting stent compared with a paclitaxel-eluting stent in patients with de novo coronary lesions: 2-year follow-up from the ENDEAVOR IV trial (Randomized, Controlled Trial of the Medtronic Endeavor Drug [ABT-578] Eluting Coronary Stent System Versus the Taxus Paclitaxel-Eluting Coronary Stent System in De Novo Native Coronary Artery Lesions). JACC Cardiovasc Interv. 12/ 2009;2(12):1208-1218.

270. Leroux EJ, Morton JM, Rivas H. Increasing access to specialty surgical care: application of a new resource allocation model to bariatric surgery. Ann Surg. 08/ 2014;260(2):274-278.

271. Letterstal A, Forsberg C, Olofsson P, Wahlberg E. Risk attitudes to treatment among patients with severe intermittent claudication. J Vasc Surg. 05/ 2008;47(5):988-994.

272. Li CS, Bhandari M. Cost-effectiveness of unicompartmental knee arthroplasty, high tibial osteotomy, and KineSpring® Knee Implant System for unicompartmental osteoarthritis of the knee. J Long Term Eff Med Implants. / 2013;23(2-3):189-198.

273. Li CS, Seeger T, Auhuber TC, Bhandari M. Cost-effectiveness and economic impact of the KineSpring ® Knee Implant System in the treatment for knee osteoarthritis. Knee Surg Sports Traumatol Arthrosc. 11/ 2013;21(11):2629-2637.

274. Li EY, Tham CC, Chi SC, Lam DS. Cost-effectiveness of treating normal tension glaucoma. Invest Ophthalmol Vis Sci. 05/ 2013;54(5):3394-3399.

275. Lier D, Ross S, Tang S, Robert M, Jacobs P. Trans-obturator tape compared with tension-free vaginal tape in the surgical treatment of stress urinary incontinence: a cost utility analysis. BJOG. 04/ 2011;118(5):550-556.

276. Ljungman D, Hyltander A, Lundholm K. Cost-utility estimations of palliative care in patients with pancreatic adenocarcinoma: a retrospective analysis. World J Surg. 08/ 2013;37(8):1883-1891.

277. Ljungman D, Lundholm K, Hyltander A. Cost-utility estimation of surgical treatment of pancreatic carcinoma aimed at cure. World J Surg. 03/ 2011;35(3):662-670.

278. Lois N, Burr J, Norrie J, Vale L, Cook J, McDonald A. Clinical and cost-effectiveness of internal limiting membrane peeling for patients with idiopathic full thickness macular hole. Protocol for a randomised controlled trial: FILMS (Full-thickness Macular Hole and Internal Limiting Membrane Peeling Study). Trials. / 2008;9:61-61.

279. Long EF, Swain GW, Mangi AA. Comparative survival and cost-effectiveness of advanced therapies for end-stage heart failure. Circ Heart Fail. 05/ 2014;7(3):470-478.

280. Losina E, Walensky RP, Kessler CL, et al. Cost-effectiveness of total knee arthroplasty in the United States: patient risk and hospital volume. Arch Intern Med. 06/22 2009;169(12):1113-1121.

281. Luebke T, Brunkwall J. Cost-effectiveness of endovascular versus open repair of acute complicated type B aortic dissections. J Vasc Surg. 05/ 2014;59(5):1247-1255.

282. Lyth J, Andersson S-O, Andren O, Johansson J-E, Carlsson P, Shahsavar N. A decision support model for cost-effectiveness of radical prostatectomy in localized prostate cancer. Scand J Urol Nephrol. 02/ 2012;46(1):19-25.

283. Ma Y, Ying X, Zou H, et al. Rhegmatogenous retinal detachment surgery in elderly people over 70 years old: visual acuity, quality of life, and cost-utility values. PLoS One. / 2014;9(10).

284. Ma Y, Ying X, Zou H, et al. Cost-utility Analysis of Rhegmatogenous Retinal Detachment Surgery in Shanghai, China. Ophthalmic Epidemiol. 02/ 2015;22(1):13-19.

285. Magee WP, Vander Burg R, Hatcher KW. Cleft lip and palate as a cost-effective health care treatment in the developing world. World J Surg. 03/ 2010;34(3):420-427.

286. Magnuson EA, Farkouh ME, Fuster V, et al. Cost-effectiveness of percutaneous coronary intervention with drug eluting stents versus bypass surgery for patients with diabetes mellitus and multivessel coronary artery disease: results from the FREEDOM trial. Circulation. 02/19 2013;127(7):820-831.

287. Mahle WT, Ianucci G, Vincent RN, Kanter KR. Costs associated with ventricular assist device use in children. Ann Thorac Surg. 11/ 2008;86(5):1592-1597.

288. Mahoney EM, Greenberg D, Lavelle TA, et al. Costs and cost-effectiveness of carotid stenting versus endarterectomy for patients at increased surgical risk: results from the SAPPHIRE trial. Catheter Cardiovasc Interv. 03/01 2011;77(4):463-472.

289. Maklin S, Malmivaara A, Linna M, Victorzon M, Koivukangas V, Sintonen H. Cost-utility of bariatric surgery for morbid obesity in Finland. Br J Surg. 10/ 2011;98(10):1422-1429.

290. Malmivaara K, Juvela S, Hernesniemi J, Lappalainen J, Siironen J. Health-related quality of life and cost-effectiveness of treatment in subarachnoid haemorrhage. Eur J Neurol. 11/ 2012;19(11):1455-1461.

291. Malmivaara K, Kivisaari R, Hernesniemi J, Siironen J. Cost-effectiveness of decompressive craniectomy in traumatic brain injuries. Eur J Neurol. 04/ 2011;18(4):656-662.

292. Malmivaara K, Ohman J, Kivisaari R, Hernesniemi J, Siironen J. Cost-effectiveness of decompressive craniectomy in non-traumatic neurological emergencies. Eur J Neurol. 03/ 2011;18(3):402-409.

293. Manca A, Sculpher MJ, Ward K, Hilton P. A cost-utility analysis of tension-free vaginal tape versus colposuspension for primary urodynamic stress incontinence. BJOG. 03/ 2003;110(3):255-262.

294. Manchikanti L, Helm S, Pampati V, Racz GB. Cost Utility Analysis of Percutaneous Adhesiolysis in Managing Pain of Post-lumbar Surgery Syndrome and Lumbar Central Spinal Stenosis. Pain Pract. 03/26 2014.

295. Manns B, Meltzer D, Taub K, Donaldson C. Illustrating the impact of including future costs in economic evaluations: an application to end-stage renal disease care. Health Econ. 11/ 2003;12(11):949-958.

296. Marcacci M, Zaffagnini S, Li CS, Bhandari M. Cost-effectiveness and economic impact of the KineSpring® Knee Implant System in the treatment of knee osteoarthritis in Italy. J Long Term Eff Med Implants. / 2013;23(2-3):211-222.

297. Marciante KD, Veenstra DL, Lipsky BA, Saint S. Which antimicrobial impregnated central venous catheter should we use? Modeling the costs and outcomes of antimicrobial catheter use. Am J Infect Control. 02/ 2003;31(1):1-8.

298. Marinelli M, Soccetti A, Panfoli N, de Palma L. Cost-effectiveness of cemented versus cementless total hip arthroplasty. A Markov decision analysis based on implant cost. J Orthop Traumatol. 03/ 2008;9(1):23-28.

299. Mark DB, Pan W, Clapp-Channing NE, et al. Quality of life after late invasive therapy for occluded arteries. N Engl J Med. 02/19 2009;360(8):774-783.

300. Marseille E. Cost-effectiveness of cataract surgery in a public health eye care programme in Nepal. Bull World Health Organ. / 1996;74(3):319-324.

301. Martino J, Gomez E, Bilbao JL, Duenas JC, Vazquez-Barquero A. Cost-utility of maximal safe resection of WHO grade II gliomas within eloquent areas. Acta Neurochir (Wien). 01/ 2013;155(1):41-50.

302. Matas AJ, Schnitzler M. Payment for living donor (vendor) kidneys: a cost-effectiveness analysis. Am J Transplant. 02/ 2004;4(2):216-221.

303. Mather RC, Hettrich CM, Dunn WR, et al. Cost-Effectiveness Analysis of Early Reconstruction Versus Rehabilitation and Delayed Reconstruction for Anterior Cruciate Ligament Tears. Am J Sports Med. 05/06 2014;42(7):1583-1591.

304. Mather RC, Hug KT, Orlando LA, et al. Economic evaluation of access to musculoskeletal care: the case of waiting for total knee arthroplasty. BMC Musculoskelet Disord. / 2014;15:22-22.

305. Mather RC, Koenig L, Acevedo D, et al. The societal and economic value of rotator cuff repair. J Bone Joint Surg Am. 11/20 2013;95(22):1993-2000.

306. Mather RC, Koenig L, Kocher MS, et al. Societal and economic impact of anterior cruciate ligament tears. J Bone Joint Surg Am. 10/02 2013;95(19):1751-1759.

307. Mather RC, Watters TS, Orlando LA, Bolognesi MP, Moorman CT. Cost effectiveness analysis of hemiarthroplasty and total shoulder arthroplasty. J Shoulder Elbow Surg. 04/ 2010;19(3):325-334.

308. Maud A, Lakshminarayan K, Suri MFK, Vazquez G, Lanzino G, Qureshi AI. Cost-effectiveness analysis of endovascular versus neurosurgical treatment for ruptured intracranial aneurysms in the United States. J Neurosurg. 05/ 2009;110(5):880-886.

309. McCord C, Chowdhury Q. A cost effective small hospital in Bangladesh: what it can mean for emergency obstetric care. Int J Gynaecol Obstet. 04/ 2003;81(1):83-92.

310. McEwen LN, Coelho RB, Baumann LM, Bilik D, Nota-Kirby B, Herman WH. The cost, quality of life impact, and cost-utility of bariatric surgery in a managed care population. Obes Surg. 07/ 2010;20(7):919-928.

311. McKay A, Kutnikoff T, Taylor M. A cost-utility analysis of treatments for malignant liver tumours: a pilot project. HPB (Oxford). / 2007;9(1):42-51.

312. McKenna C, Palmer S, Rodgers M, et al. Cost-effectiveness of radiofrequency catheter ablation for the treatment of atrial fibrillation in the United Kingdom. Heart. 04/ 2009;95(7):542-549.

313. McKenzie L, Vale L, Stearns S, McCormack K. Metal on metal hip resurfacing arthroplasty. An economic analysis. Eur J Health Econ. / 2003;4(2):122-129.

314. Mealing S, Feldman T, Eaton J, Singh M, Scott DA. EVEREST II high risk study based UK cost-effectiveness analysis of MitraClip® in patients with severe mitral regurgitation ineligible for conventional repair/replacement surgery. J Med Econ. 11/ 2013;16(11):1317-1326.

315. Mehta M, Noyes W, Craig B, et al. A cost-effectiveness and cost-utility analysis of radiosurgery vs. resection for single-brain metastases. Int J Radiat Oncol Biol Phys. 09/01 1997;39(2):445-454.

316. Mendeloff J, Ko K, Roberts MS, Byrne M, Dew MA. Procuring organ donors as a health investment: how much should we be willing to spend? Transplantation. 12/27 2004;78(12):1704-1710.

317. Mendonca L, Perelman J, Rodrigues V, Fragata J. Cost-effectiveness of lung transplantation and its evolution: the Portuguese case. Eur J Health Econ. 09/ 2014;15(7):767-772.

318. Menn P, Leidl R, Holle R. A lifetime Markov model for the economic evaluation of chronic obstructive pulmonary disease. Pharmacoeconomics. 09/01 2012;30(9):825-840.

319. Mennemeyer ST, Owsley C, McGwin G. Reducing older driver motor vehicle collisions via earlier cataract surgery. Accid Anal Prev. 12/ 2013;61:203-211.

320. Michaels JA, Campbell WB, Brazier JE, et al. Randomised clinical trial, observational study and assessment of cost-effectiveness of the treatment of varicose veins (REACTIV trial). Health Technol Assess. 04/ 2006;10(13):1-196.

321. Miller JD, Malthaner RA, Goldsmith CH, et al. A randomized clinical trial of lung volume reduction surgery versus best medical care for patients with advanced emphysema: a two-year study from Canada. Ann Thorac Surg. 01/ 2006;81(1):314-320.

322. Montesino-Semper MF, Jimenez-Calvo JM, Cabases JM, Sanchez-Iriso E, Hualde-Alfaro A, Garcia-Garcia D. Cost-effectiveness analysis of the surgical treatment of female urinary incontinence using slings and meshes. Eur J Obstet Gynecol Reprod Biol. 11/ 2013;171(1):180-186.

323. Moon W, Perry H, Baek R-M. Is international volunteer surgery for cleft lip and cleft palate a cost-effective and justifiable intervention? A case study from East Asia. World J Surg. 12/ 2012;36(12):2819-2830.

324. Moreno SG, Novielli N, Cooper NJ. Cost-effectiveness of the implantable HeartMate II left ventricular assist device for patients awaiting heart transplantation. J Heart Lung Transplant. 05/ 2012;31(5):450-458.

325. Morimoto T, Shimbo T, Noguchi Y, et al. Effects of timing of thoracoscopic surgery for primary spontaneous pneumothorax on prognosis and costs. Am J Surg. 06/ 2004;187(6):767-774.

326. Moro PL, Budke CM, Schantz PM, Vasquez J, Santivanez SJ, Villavicencio J. Economic impact of cystic echinococcosis in peru. PLoS Negl Trop Dis. 05/ 2011;5(5).

327. Morris S, Gurusamy KS, Patel N, Davidson BR. Cost-effectiveness of early laparoscopic cholecystectomy for mild acute gallstone pancreatitis. Br J Surg. 06/ 2014;101(7):828-835.

328. Morton RL, Howard K, Thompson JF. The cost-effectiveness of sentinel node biopsy in patients with intermediate thickness primary cutaneous melanoma. Ann Surg Oncol. 04/ 2009;16(4):929-940.

329. Mota REM. Cost-effectiveness analysis of early versus late total hip replacement in Italy. Value Health. / 2013;16(2):267-279.

330. Mueller ER, Kenton K, Tarnay C, et al. Abdominal Colpopexy: Comparison of Endoscopic Surgical Strategies (ACCESS). Contemp Clin Trials. 09/ 2012;33(5):1011-1018.

331. Mutinga N, Brennan DC, Schnitzler MA. Consequences of eliminating HLA-B in deceased donor kidney allocation to increase minority transplantation. Am J Transplant. 05/ 2005;5(5):1090-1098.

332. Naeim A, Keeler EB, Gutierrez PR, Wilson MR, Reuben D, Mangione CM. Is cataract surgery cost-effective among older patients with a low predicted probability for improvement in reported visual functioning? Med Care. 11/ 2006;44(11):982-989.

333. Narayan R, Perkins RM, Berbano EP, et al. Parathyroidectomy versus cinacalcet hydrochloride-based medical therapy in the management of hyperparathyroidism in ESRD: a cost utility analysis. Am J Kidney Dis. 06/ 2007;49(6):801-813.

334. Nathoe HM, van Dijk D, Jansen EWL, et al. A comparison of on-pump and off-pump coronary bypass surgery in low-risk patients. N Engl J Med. 01/30 2003;348(5):394-402.

335. Navarro Espigares JL, Hernandez Torres E. Cost-outcome analysis of joint replacement: evidence from a Spanish public hospital. Gac Sanit. / 2008;22(4):337-343.

336. Neubauer AS, Liakopoulos S, van Meurs JC, Kirchhof B. Cost-effectiveness of autologous retinal pigment epithelium and choroid translocation in neovascular AMD. Int J Ophthalmol. / 2010;3(3):228-233.

337. Neyt M, Van Brabandt H, Devriese S, Van De Sande S. A cost-utility analysis of transcatheter aortic valve implantation in Belgium: focusing on a well-defined and identifiable population. BMJ Open. / 2012;2(3).

338. Neyt M, Van den Bruel A, Smit Y, et al. Cost-effectiveness of continuous-flow left ventricular assist devices. Int J Technol Assess Health Care. 07/ 2013;29(3):254-260.

339. Nguyen GC, Frick KD, Dassopoulos T. Medical decision analysis for the management of unifocal, flat, low-grade dysplasia in ulcerative colitis. Gastrointest Endosc. 06/ 2009;69(7):1299-1310.

340. Niens LM, Zelle SG, Gutierrez-Delgado C, et al. Cost-effectiveness of breast cancer control strategies in Central America: the cases of Costa Rica and Mexico. PLoS One. / 2014;9(4).

341. Northup PG, Abecassis MM, Englesbe MJ, et al. Addition of adult-to-adult living donation to liver transplant programs improves survival but at an increased cost. Liver Transpl. 02/ 2009;15(2):148-162.

342. Norum J, Olsen JA. A cost-effectiveness approach to the Norwegian follow-up programme in colorectal cancer. Ann Oncol. 11/ 1997;8(11):1081-1087.

343. Norum J, Olsen JA, Wist EA. Lumpectomy or mastectomy? Is breast conserving surgery too expensive? Breast Cancer Res Treat. 08/ 1997;45(1):7-14.

344. Nouso K, Tanaka H, Uematsu S, et al. Cost-effectiveness of the surveillance program of hepatocellular carcinoma depends on the medical circumstances. J Gastroenterol Hepatol. 03/ 2008;23(3):437-444.

345. O'Neill C, Archbold SM, O'Donoghue GM, McAlister DA, Nikolopoulos TP. Indirect costs, cost-utility variations and the funding of paediatric cochlear implantation. Int J Pediatr Otorhinolaryngol. 04/06 2001;58(1):53-57.

346. O'Neill C, O'Donoghue GM, Archbold SM, Normand C. A cost-utility analysis of pediatric cochlear implantation. Laryngoscope. 01/ 2000;110(1):156-160.

347. O'Shea K, Bale E, Murray P. Cost analysis of primary total hip replacement. Ir Med J. 06/ 2002;95(6):177-180.

348. Oddershede L, Andreasen JJ, Brocki BC, Ehlers L. Economic evaluation of endoscopic versus open vein harvest for coronary artery bypass grafting. Ann Thorac Surg. 04/ 2012;93(4):1174-1180.

349. Oddershede L, Riahi S, Nielsen JC, Hjortshoj S, Andersen HR, Ehlers L. Health economic evaluation of single-lead atrial pacing vs. dual-chamber pacing in sick sinus syndrome. Europace. 06/ 2014;16(6):866-872.

350. Oestergaard LG, Christensen FB, Nielsen CV, Bunger CE, Fruensgaard S, Sogaard R. Early versus late initiation of rehabilitation after lumbar spinal fusion: economic evaluation alongside a randomized controlled trial. Spine (Phila Pa 1976). 11/01 2013;38(23):1979-1985.

351. Osnes-Ringen H, Kvamme MK, Kristiansen IS, et al. Cost-effectiveness analyses of elective orthopaedic surgical procedures in patients with inflammatory arthropathies. Scand J Rheumatol. 03/ 2011;40(2):108-115.

352. Ouwens JP, van Enckevort PJ, TenVergert EM, et al. The cost effectiveness of lung transplantation compared with that of heart and liver transplantation in the Netherlands. Transpl Int. 02/ 2003;16(2):123-127.

353. Pace KT, Dyer SJ, Phan V, et al. Laparoscopic v open donor nephrectomy: a cost-utility analysis of the initial experience at a tertiary-care center. J Endourol. 09/ 2002;16(7):495-508.

354. Pace KT, Dyer SJ, Phan V, et al. Laparoscopic versus open donor nephrectomy. Surg Endosc. 01/ 2003;17(1):134-142.

355. Palmqvist E, Larsson K, Anell A, Hjalmarsson C. Prospective study of pain, quality of life and the economic impact of open inguinal hernia repair. Br J Surg. 10/ 2013;100(11):1483-1488.

356. Pandharipande PV, Gervais DA, Mueller PR, Hur C, Gazelle GS. Radiofrequency ablation versus nephron-sparing surgery for small unilateral renal cell carcinoma: cost-effectiveness analysis. Radiology. 07/ 2008;248(1):169-178.

357. Parker SL, Adogwa O, Bydon A, Cheng J, McGirt MJ. Cost-effectiveness of minimally invasive versus open transforaminal lumbar interbody fusion for degenerative spondylolisthesis associated low-back and leg pain over two years. World Neurosurg. 07/ 2012;78(1-2):178-184.

358. Parkinson B, Goodall S, Thavaneswaran P. Cost-effectiveness of lumbar artificial intervertebral disc replacement: driven by the choice of comparator. ANZ J Surg. 09/ 2013;83(9):669-675.

359. Patel D, Terrault NA, Yao FY, Bass NM, Ladabaum U. Cost-effectiveness of hepatocellular carcinoma surveillance in patients with hepatitis C virus-related cirrhosis. Clin Gastroenterol Hepatol. 01/ 2005;3(1):75-84.

360. Patel ST, Haser PB, Bush HL, Kent KC. The cost-effectiveness of endovascular repair versus open surgical repair of abdominal aortic aneurysms: A decision analysis model. J Vasc Surg. 06/ 1999;29(6):958-972.

361. Patil CG, Long EF, Lansberg MG. Cost-effectiveness analysis of mechanical thrombectomy in acute ischemic stroke. J Neurosurg. 03/ 2009;110(3):508-513.

362. Pearson AM, Tosteson ANA, Koval KJ, et al. Is surgery for displaced, midshaft clavicle fractures in adults cost-effective? Results based on a multicenter randomized, controlled trial. J Orthop Trauma. 07/ 2010;24(7):426-433.

363. Pennington M, Grieve R, Sekhon JS, Gregg P, Black N, van der Meulen JH. Cemented, cementless, and hybrid prostheses for total hip replacement: cost effectiveness analysis. BMJ. / 2013;346.

364. Perovic S, Jankovic S. Renal transplantation vs hemodialysis: cost-effectiveness analysis. Vojnosanit Pregl. 08/ 2009;66(8):639-644.

365. Pickard JD, Bailey S, Sanderson H, Rees M, Garfield JS. Steps towards cost-benefit analysis of regional neurosurgical care. BMJ. 09/29 1990;301(6753):629-635.

366. Pickett CA, Villines TC, Ferguson MA, Hulten EA. Cost effectiveness of percutaneous closure versus medical therapy for cryptogenic stroke in patients with a patent foramen ovale. Am J Cardiol. 11/15 2014;114(10):1584-1589.

367. Pineda R, Denevich S, Lee WC, Waycaster C, Pashos CL. Economic evaluation of toric intraocular lens: a short- and long-term decision analytic model. Arch Ophthalmol. 07/ 2010;128(7):834-840.

368. Plunkett BA, Grobman WA. Elective cesarean delivery to prevent perinatal transmission of hepatitis C virus: a cost-effectiveness analysis. Am J Obstet Gynecol. 09/ 2004;191(3):998-991003.

369. Poenaru D. Getting the job done: analysis of the impact and effectiveness of the SmileTrain program in alleviating the global burden of cleft disease. World J Surg. 07/ 2013;37(7):1562-1570.

370. Poh CF, Durham JS, Brasher PM, et al. Canadian Optically-guided approach for Oral Lesions Surgical (COOLS) trial: study protocol for a randomized controlled trial. BMC Cancer. / 2011;11:462-462.

371. Poley MJ, Stolk EA, Langemeijer RA, Molenaar JC, Busschbach JJ. The cost-effectiveness of neonatal surgery and subsequent treatment for congenital anorectal malformations. J Pediatr Surg. 10/ 2001;36(10):1471-1478.

372. Poley MJ, Stolk EA, Tibboel D, Molenaar JC, Busschbach JJv. The cost-effectiveness of treatment for congenital diaphragmatic hernia. J Pediatr Surg. 09/ 2002;37(9):1245-1252.

373. Pollock RF, Muduma G, Valentine WJ. Evaluating the cost-effectiveness of laparoscopic adjustable gastric banding versus standard medical management in obese patients with type 2 diabetes in the UK. Diabetes Obes Metab. 02/ 2013;15(2):121-129.

374. Polsky D, Mandelblatt JS, Weeks JC, et al. Economic evaluation of breast cancer treatment: considering the value of patient choice. J Clin Oncol. 03/15 2003;21(6):1139-1146.

375. Porter PJ, Shin AY, Detsky AS, Lefaive L, Wallace MC. Surgery versus stereotactic radiosurgery for small, operable cerebral arteriovenous malformations: a clinical and cost comparison. Neurosurgery. 10/ 1997;41(4):757-764.

376. Postma EL, Koffijberg H, Verkooijen HM, Witkamp AJ, van den Bosch MAAJ, van Hillegersberg R. Cost-effectiveness of radioguided occult lesion localization (ROLL) versus wire-guided localization (WGL) in breast conserving surgery for nonpalpable breast cancer: results from a randomized controlled multicenter trial. Ann Surg Oncol. 07/ 2013;20(7):2219-2226.

377. Poulose BK, Speroff T, Holzman MD. Optimizing choledocholithiasis management: a cost-effectiveness analysis. Arch Surg. 01/ 2007;142(1):43-48.

378. Prabhu SS, Kaakeh R, Sugar A, Smith DG, Shtein RM. Comparative cost-effectiveness analysis of descemet stripping automated endothelial keratoplasty versus penetrating keratoplasty in the United States. Am J Ophthalmol. 01/ 2013;155(1):45-53.

379. Prieto-Diaz-Chavez E, Medina-Chavez JL, Anaya-Prado R. A cost-effectiveness analysis of tension-free versus shouldice inguinal hernia repair: a randomized double-blind clinical trial. Hernia. 06/ 2009;13(3):233-238.

380. Prinssen M, Buskens E, de Jong SE, et al. Cost-effectiveness of conventional and endovascular repair of abdominal aortic aneurysms: results of a randomized trial. J Vasc Surg. 11/ 2007;46(5):883-890.

381. Puri V, Crabtree TD, Kymes S, et al. A comparison of surgical intervention and stereotactic body radiation therapy for stage I lung cancer in high-risk patients: a decision analysis. J Thorac Cardiovasc Surg. 02/ 2012;143(2):428-436.

382. Puri V, Pyrdeck TL, Crabtree TD, et al. Treatment of malignant pleural effusion: a cost-effectiveness analysis. Ann Thorac Surg. 08/ 2012;94(2):374-379.

383. Quenneville SP, Xie X, Brophy JM. The cost-effectiveness of Maze procedures using ablation techniques at the time of mitral valve surgery. Int J Technol Assess Health Care. 10/ 2009;25(4):485-496.

384. Rampersaud YR, Tso P, Walker KR, et al. Comparative outcomes and cost-utility following surgical treatment of focal lumbar spinal stenosis compared with osteoarthritis of the hip or knee: part 2--estimated lifetime incremental cost-utility ratios. Spine J. 02/01 2014;14(2):244-254.

385. Ramsey SD, Berry K, Etzioni R, Kaplan RM, Sullivan SD, Wood DE. Cost effectiveness of lung-volume-reduction surgery for patients with severe emphysema. N Engl J Med. 05/22 2003;348(21):2092-2102.

386. Ramsey SD, Blough DK, Sullivan SD. A forensic evaluation of the National Emphysema Treatment Trial using the expected value of information approach. Med Care. 05/ 2008;46(5):542-548.

387. Ramsey SD, Shroyer AL, Sullivan SD, Wood DE. Updated evaluation of the cost-effectiveness of lung volume reduction surgery. Chest. 03/ 2007;131(3):823-832.

388. Ramsey SD, Sullivan SD, Kaplan RM. Cost-effectiveness of lung volume reduction surgery. Proc Am Thorac Soc. 05/01 2008;5(4):406-411.

389. Randolph AG, Washington AE, Prober CG. Cesarean delivery for women presenting with genital herpes lesions. Efficacy, risks, and costs. JAMA. 07/07 1993;270(1):77-82.

390. Rasanen P, Krootila K, Sintonen H, et al. Cost-utility of routine cataract surgery. Health Qual Life Outcomes. / 2006;4:74-74.

391. Rasanen P, Ohman J, Sintonen H, et al. Cost-utility analysis of routine neurosurgical spinal surgery. J Neurosurg Spine. 09/ 2006;5(3):204-209.

392. Rasanen P, Paavolainen P, Sintonen H, et al. Effectiveness of hip or knee replacement surgery in terms of quality-adjusted life years and costs. Acta Orthop. 02/ 2007;78(1):108-115.

393. Ratcliffe J, Buxton M, Young T, Longworth L. Determining priority for liver transplantation: a comparison of cost per QALY and discrete choice experiment-generated public preferences. Appl Health Econ Health Policy. / 2005;4(4):249-255.

394. Rein DB, Wittenborn JS, Lee PP, et al. The cost-effectiveness of routine office-based identification and subsequent medical treatment of primary open-angle glaucoma in the United States. Ophthalmology. 05/ 2009;116(5):823-832.

395. Remak E, Manson S, Hutton J, Brasseur P, Olivier E, Gershlick A. Cost-effectiveness of the Endeavor stent in de novo native coronary artery lesions updated with contemporary data. EuroIntervention. 02/ 2010;5(7):826-832.

396. Renfree KJ, Hattrup SJ, Chang Y-HH. Cost utility analysis of reverse total shoulder arthroplasty. J Shoulder Elbow Surg. 12/ 2013;22(12):1656-1661.

397. Reynolds MR, Zimetbaum P, Josephson ME, Ellis E, Danilov T, Cohen DJ. Cost-effectiveness of radiofrequency catheter ablation compared with antiarrhythmic drug therapy for paroxysmal atrial fibrillation. Circ Arrhythm Electrophysiol. 08/ 2009;2(4):362-369.

398. Ribaric G, Kofler J, Jayne DG. Stapled hemorrhoidopexy, an innovative surgical procedure for hemorrhoidal prolapse: cost-utility analysis. Croat Med J. 08/15 2011;52(4):497-504.

399. Roberts A, Habibi M, Frick KD. Cost-effectiveness of contralateral prophylactic mastectomy for prevention of contralateral breast cancer. Ann Surg Oncol. 07/ 2014;21(7):2209-2217.

400. Rogers JG, Bostic RR, Tong KB, Adamson R, Russo M, Slaughter MS. Cost-effectiveness analysis of continuous-flow left ventricular assist devices as destination therapy. Circ Heart Fail. 01/ 2012;5(1):10-16.

401. Roos JB, Doshi SN, Konorza T, et al. The cost-effectiveness of a new percutaneous ventricular assist device for high-risk PCI patients: mid-stage evaluation from the European perspective. J Med Econ. / 2013;16(3):381-390.

402. Rosas SE, Feldman HI. Synthetic vascular hemodialysis access versus native arteriovenous fistula: a cost-utility analysis. Ann Surg. 01/ 2012;255(1):181-186.

403. Routh JC, Laufer MR, Cannon GM, Diamond DA, Gargollo PC. Management strategies for Mayer-Rokitansky-Kuster-Hauser related vaginal agenesis: a cost-effectiveness analysis. J Urol. 11/ 2010;184(5):2116-2121.

404. Rudmik L, Starreveld YP, Vandergrift WA, Banglawala SM, Soler ZM. Cost-effectiveness of the endoscopic versus microscopic approach for pituitary adenoma resection. Laryngoscope. 01/ 2015;125(1):16-24.

405. Ruiz D, Koenig L, Dall TM, et al. The direct and indirect costs to society of treatment for end-stage knee osteoarthritis. J Bone Joint Surg Am. 08/21 2013;95(16):1473-1480.

406. Saariniemi KMM, Kuokkanen HOM, Rasanen P, Sintonen H, Tukiainen EJ. The cost utility of reduction mammaplasty at medium-term follow-up: a prospective study. J Plast Reconstr Aesthet Surg. 01/ 2012;65(1):17-21.

407. Sagmeister M, Mullhaupt B, Kadry Z, Kullak-Ublick GA, Clavien PA, Renner EL. Cost-effectiveness of cadaveric and living-donor liver transplantation. Transplantation. 02/27 2002;73(4):616-622.

408. Salem L, Devlin A, Sullivan SD, Flum DR. Cost-effectiveness analysis of laparoscopic gastric bypass, adjustable gastric banding, and nonoperative weight loss interventions. Surg Obes Relat Dis. / 2008;4(1):26-32.

409. Salomon JA, Carvalho N, Gutierrez-Delgado C, et al. Intervention strategies to reduce the burden of non-communicable diseases in Mexico: cost effectiveness analysis. BMJ. / 2012;344.

410. Samuelson EM, Brown DE. Cost-effectiveness analysis of autologous chondrocyte implantation: a comparison of periosteal patch versus type I/III collagen membrane. Am J Sports Med. 06/ 2012;40(6):1252-1258.

411. Sangchan A, Chaiyakunapruk N, Supakankunti S, Pugkhem A, Mairiang P. Cost utility analysis of endoscopic biliary stent in unresectable hilar cholangiocarcinoma: decision analytic modeling approach. Hepatogastroenterology. / 2014;61(133):1175-1181.

412. Sansom SL, Prabhu VS, Hutchinson AB, et al. Cost-effectiveness of newborn circumcision in reducing lifetime HIV risk among U.S. males. PLoS One. / 2010;5(1).

413. Sarasin FP, Majno PE, Llovet JM, Bruix J, Mentha G, Hadengue A. Living donor liver transplantation for early hepatocellular carcinoma: A life-expectancy and cost-effectiveness perspective. Hepatology. 05/ 2001;33(5):1073-1079.

414. Schackman BR, Oneda K, Goldie SJ. The cost-effectiveness of elective Cesarean delivery to prevent hepatitis C transmission in HIV-coinfected women. AIDS. 09/03 2004;18(13):1827-1834.

415. Schermerhorn ML, Birkmeyer JD, Gould DA, Cronenwett JL. Cost-effectiveness of surgery for small abdominal aortic aneurysms on the basis of data from the United Kingdom small aneurysm trial. J Vasc Surg. 02/ 2000;31(2):217-226.

416. Schmier JK, Halevi M, Maislin G, Ong K. Comparative cost effectiveness of Coflex® interlaminar stabilization versus instrumented posterolateral lumbar fusion for the treatment of lumbar spinal stenosis and spondylolisthesis. Clinicoecon Outcomes Res. / 2014;6:125-131.

417. Sculpher M. A cost-utility analysis of abdominal hysterectomy versus transcervical endometrial resection for the surgical treatment of menorrhagia. Int J Technol Assess Health Care. / 1998;14(2):302-319.

418. Sears ED, Shin R, Prosser LA, Chung KC. Economic analysis of revision amputation and replantation treatment of finger amputation injuries. Plast Reconstr Surg. 04/ 2014;133(4):827-840.

419. Seidler AM, Bramlette TB, Washington CV, Szeto H, Chen SC. Mohs versus traditional surgical excision for facial and auricular nonmelanoma skin cancer: an analysis of cost-effectiveness. Dermatol Surg. 11/ 2009;35(11):1776-1787.

420. Sejean K, Calmus S, Durand-Zaleski I, et al. Surgery versus medical follow-up in patients with asymptomatic primary hyperparathyroidism: a decision analysis. Eur J Endocrinol. 12/ 2005;153(6):915-927.

421. Seklehner S, Laudano MA, Te AE, Kaplan SA, Chughtai B, Lee RK. A cost-effectiveness analysis of retropubic midurethral sling versus transobturator midurethral sling for female stress urinary incontinence. Neurourol Urodyn. 11/ 2014;33(8):1186-1192.

422. Semenov YR, Yeh ST, Seshamani M, et al. Age-dependent cost-utility of pediatric cochlear implantation. Ear Hear. / 2013;34(4):402-412.

423. Sgourakis G, Dedemadi G, Gockel I, et al. Laparoscopic totally extraperitoneal versus open preperitoneal mesh repair for inguinal hernia recurrence: a decision analysis based on net health benefits. Surg Endosc. 07/ 2013;27(7):2526-2541.

424. Shaheen NJ, Inadomi JM, Overholt BF, Sharma P. What is the best management strategy for high grade dysplasia in Barrett's oesophagus? A cost effectiveness analysis. Gut. 12/ 2004;53(12):1736-1744.

425. Sharifi E, Sharifi H, Morshed S, Bozic K, Diab M. Cost-effectiveness analysis of periacetabular osteotomy. J Bone Joint Surg Am. 07/ 2008;90(7):1447-1456.

426. Sharma S, Brown GC, Brown MM, Hollands H, Shah GK. The cost-effectiveness of grid laser photocoagulation for the treatment of diabetic macular edema: results of a patient-based cost-utility analysis. Curr Opin Ophthalmol. 06/ 2000;11(3):175-179.

427. Sharma S, Hollands H, Brown GC, Brown MM, Shah GK, Sharma SM. The cost-effectiveness of early vitrectomy for the treatment of vitreous hemorrhage in diabetic retinopathy. Curr Opin Ophthalmol. 06/ 2001;12(3):230-234.

428. Sharples L, Buxton M, Caine N, et al. Evaluation of the ventricular assist device programme in the UK. Health Technol Assess. 11/ 2006;10(48):1-119.

429. Sharples LD, Dyer M, Cafferty F, et al. Cost-effectiveness of ventricular assist device use in the United Kingdom: results from the evaluation of ventricular assist device programme in the UK (EVAD-UK). J Heart Lung Transplant. 11/ 2006;25(11):1336-1343.

430. Shauver MJ, Clapham PJ, Chung KC. An economic analysis of outcomes and complications of treating distal radius fractures in the elderly. J Hand Surg Am. 12/ 2011;36(12):1912-1918.

431. Shiga T, Apfel CC, Wajima Zi, Ohe Y. Influence of intraoperative conversion from off-pump to on-pump coronary artery bypass grafting on costs and quality of life: a cost-effectiveness analysis. J Cardiothorac Vasc Anesth. 12/ 2007;21(6):793-799.

432. Shillcutt SD, Clarke MG, Kingsnorth AN. Cost-effectiveness of groin hernia surgery in the Western Region of Ghana. Arch Surg. 10/ 2010;145(10):954-961.

433. Shillcutt SD, Sanders DL, Teresa Butron-Vila M, Kingsnorth AN. Cost-effectiveness of inguinal hernia surgery in northwestern Ecuador. World J Surg. 01/ 2013;37(1):32-41.

434. Shrive FM, Ghali WA, Johnson JA, Donaldson C, Manns BJ. Use of the U.S. and U.K. scoring algorithm for the EuroQol-5D in an economic evaluation of cardiac care. Med Care. 03/ 2007;45(3):269-273.

435. Simons CT, Cipriano LE, Shah RU, Garber AM, Owens DK, Hlatky MA. Transcatheter aortic valve replacement in nonsurgical candidates with severe, symptomatic aortic stenosis: a cost-effectiveness analysis. Circ Cardiovasc Qual Outcomes. 07/ 2013;6(4):419-428.

436. Singh SM, Micieli A, Wijeysundera HC. Economic evaluation of percutaneous left atrial appendage occlusion, dabigatran, and warfarin for stroke prevention in patients with nonvalvular atrial fibrillation. Circulation. 06/18 2013;127(24):2414-2423.

437. Slobogean GP, Marra CA, Sadatsafavi M, Sanders DW. Is surgical fixation for stress-positive unstable ankle fractures cost effective? Results of a multicenter randomized control trial. J Orthop Trauma. 11/ 2012;26(11):652-658.

438. Slover J, Hoffman MV, Malchau H, Tosteson ANA, Koval KJ. A cost-effectiveness analysis of the arthroplasty options for displaced femoral neck fractures in the active, healthy, elderly population. J Arthroplasty. 09/ 2009;24(6):854-860.

439. Smiddy WE. Economic considerations of macular edema therapies. Ophthalmology. 09/ 2011;118(9):1827-1833.

440. Snyder RA, Moore DR, Moore DE. More donors or more delayed graft function? A cost-effectiveness analysis of DCD kidney transplantation. Clin Transplant. / 2013;27(2):289-296.

441. Soegaard R, Bunger CE, Christiansen T, Hoy K, Eiskjaer SP, Christensen FB. Circumferential fusion is dominant over posterolateral fusion in a long-term perspective: cost-utility evaluation of a randomized controlled trial in severe, chronic low back pain. Spine (Phila Pa 1976). 10/15 2007;32(22):2405-2414.

442. Song HJ, Kwon JW, Kim YJ, Oh S-H, Heo Y, Han S-M. Bariatric surgery for the treatment of severely obese patients in South Korea--is it cost effective? Obes Surg. 12/ 2013;23(12):2058-2067.

443. Song JW, Chung KC, Prosser LA. Treatment of ulnar neuropathy at the elbow: cost-utility analysis. J Hand Surg Am. 08/ 2012;37(8):1617-1629.

444. Soohoo NF, Sharifi H, Kominski G, Lieberman JR. Cost-effectiveness analysis of unicompartmental knee arthroplasty as an alternative to total knee arthroplasty for unicompartmental osteoarthritis. J Bone Joint Surg Am. 09/ 2006;88(9):1975-1982.

445. Sousa P, Perelman J, Dimitrovova K, et al. Cost-effectiveness of the endovascular repair of Abdominal Aortic Aneurysm in Portugal. Angiologia e Cirurgia Vascular. 2014;10(2):41-48.

446. Sprague S, Bhandari M. An economic evaluation of early versus delayed operative treatment in patients with closed tibial shaft fractures. Arch Orthop Trauma Surg. 07/ 2002;122(6):315-323.

447. Spronk S, Bosch JL, den Hoed PT, Veen HF, Pattynama PMT, Hunink MGM. Cost-effectiveness of endovascular revascularization compared to supervised hospital-based exercise training in patients with intermittent claudication: a randomized controlled trial. J Vasc Surg. 12/ 2008;48(6):1472-1480.

448. Stevens K, McCabe C, Jones C, Ashcroft J, Harvey S, Rowan K. The incremental cost effectiveness of withdrawing pulmonary artery catheters from routine use in critical care. Appl Health Econ Health Policy. / 2005;4(4):257-264.

449. Stewart WC, Stewart JA, Nasser QJ, Nassar QJ, Mychaskiw MA. Cost-effectiveness of treating ocular hypertension. Ophthalmology. 01/ 2008;115(1):94-98.

450. Strom O, Leonard C, Marsh D, Cooper C. Cost-effectiveness of balloon kyphoplasty in patients with symptomatic vertebral compression fractures in a UK setting. Osteoporos Int. 09/ 2010;21(9):1599-1608.

451. Stroupe KT, Lederle FA, Matsumura JS, et al. Cost-effectiveness of open versus endovascular repair of abdominal aortic aneurysm in the OVER trial. J Vasc Surg. 10/ 2012;56(4):901-909.

452. Stroupe KT, Manheim LM, Luo P, et al. Tension-free repair versus watchful waiting for men with asymptomatic or minimally symptomatic inguinal hernias: a cost-effectiveness analysis. J Am Coll Surg. 10/ 2006;203(4):458-468.

453. Su X, Zenios SA, Chertow GM. Incorporating recipient choice in kidney transplantation. J Am Soc Nephrol. 06/ 2004;15(6):1656-1663.

454. Sugar EA, Holbrook JT, Kempen JH, et al. Cost-effectiveness of fluocinolone acetonide implant versus systemic therapy for noninfectious intermediate, posterior, and panuveitis. Ophthalmology. 10/ 2014;121(10):1855-1862.

455. Sultan S, Hynes N. Clinical efficacy and cost per quality-adjusted life years of pararenal endovascular aortic aneurysm repair compared with open surgical repair. J Endovasc Ther. 04/ 2011;18(2):181-196.

456. Sultan S, Tawfick W, Hynes N. Cool excimer laser-assisted angioplasty (CELA) and tibial balloon angioplasty (TBA) in management of infragenicular arterial occlusion in critical lower limb ischemia (CLI). Vasc Endovascular Surg. 04/ 2013;47(3):179-191.

457. Summerfield AQ, Marshall DH, Barton GR, Bloor KE. A cost-utility scenario analysis of bilateral cochlear implantation. Arch Otolaryngol Head Neck Surg. 11/ 2002;128(11):1255-1262.

458. Sung JJY, Amarapurkar D, Chan HLY, et al. Treatment of chronic hepatitis B in Asia-Pacific countries: is the Asia-Pacific consensus statement being followed? Antivir Ther. / 2010;15(4):607-616.

459. Svedbom A, Alvares L, Cooper C, Marsh D, Strom O. Balloon kyphoplasty compared to vertebroplasty and nonsurgical management in patients hospitalised with acute osteoporotic vertebral compression fracture: a UK cost-effectiveness analysis. Osteoporos Int. 01/ 2013;24(1):355-367.

460. Svensjo S, Mani K, Bjorck M, Lundkvist J, Wanhainen A. Screening for abdominal aortic aneurysm in 65-year-old men remains cost-effective with contemporary epidemiology and management. Eur J Vasc Endovasc Surg. 04/ 2014;47(4):357-365.

461. Svensson J, Ghatnekar O, Lindgren A, et al. Societal value of stem cell therapy in stroke--a modeling study. Cerebrovasc Dis. / 2012;33(6):532-539.

462. Swart E, Makhni EC, Macaulay W, Rosenwasser MP, Bozic KJ. Cost-effectiveness analysis of fixation options for intertrochanteric hip fractures. J Bone Joint Surg Am. 10/01 2014;96(19):1612-1620.

463. Tadisina KK, Chopra K, Tangredi J, Thomson JG, Singh DP. Helping hands: a cost-effectiveness study of a humanitarian hand surgery mission. Plast Surg Int. / 2014;2014:921625-921625.

464. Takao H, Nojo T. Treatment of unruptured intracranial aneurysms: decision and cost-effectiveness analysis. Radiology. 09/ 2007;244(3):755-766.

465. Takao H, Nojo T, Ohtomo K. Cost-effectiveness of treatment of unruptured intracranial aneurysms in patients with a history of subarachnoid hemorrhage. Acad Radiol. 09/ 2008;15(9):1126-1132.

466. Tan JM, Macario A, Carvalho B, Druzin ML, El-Sayed YY. Cost-effectiveness of external cephalic version for term breech presentation. BMC Pregnancy Childbirth. / 2010;10:3-3.

467. Tarride J-E, Blackhouse G, De Rose G, et al. Cost-effectiveness analysis of elective endovascular repair compared with open surgical repair of abdominal aortic aneurysms for patients at a high surgical risk: A 1-year patient-level analysis conducted in Ontario, Canada. J Vasc Surg. 10/ 2008;48(4):779-787.

468. Taylor AJ, Tate D, Brandberg Y, Blomqvist L. Cost-effectiveness of reduction mammaplasty. Int J Technol Assess Health Care. / 2004;20(3):269-273.

469. Taylor RJ, Taylor RS. Spinal cord stimulation for failed back surgery syndrome: a decision-analytic model and cost-effectiveness analysis. Int J Technol Assess Health Care. / 2005;21(3):351-358.

470. Terran J, McHugh BJ, Fischer CR, et al. Surgical treatment for adult spinal deformity: projected cost effectiveness at 5-year follow-up. Ochsner J. / 2014;14(1):14-22.

471. Thaha MA, Campbell K, Bryant C, et al. Cost-effectiveness of stapled hemorrhoidopexy: A randomized comparison with ferguson closed diathermy hemorrhoidectomy. Gastroenterology. 2012;142(5):S735.

472. Thoma A, Khuthaila D, Rockwell G, Veltri K. Cost-utility analysis comparing free and pedicled TRAM flap for breast reconstruction. Microsurgery. / 2003;23(4):287-295.

473. Thoma A, Veltri K, Khuthaila D, Rockwell G, Duku E. Comparison of the deep inferior epigastric perforator flap and free transverse rectus abdominis myocutaneous flap in postmastectomy reconstruction: a cost-effectiveness analysis. Plast Reconstr Surg. 05/ 2004;113(6):1650-1661.

474. Torgerson PR, Schweiger A, Deplazes P, et al. Alveolar echinococcosis: from a deadly disease to a well-controlled infection. Relative survival and economic analysis in Switzerland over the last 35 years. J Hepatol. 07/ 2008;49(1):72-77.

475. Tosteson ANA, Lurie JD, Tosteson TD, et al. Surgical treatment of spinal stenosis with and without degenerative spondylolisthesis: cost-effectiveness after 2 years. Ann Intern Med. 12/16 2008;149(12):845-853.

476. Tosteson ANA, Skinner JS, Tosteson TD, et al. The cost effectiveness of surgical versus nonoperative treatment for lumbar disc herniation over two years: evidence from the Spine Patient Outcomes Research Trial (SPORT). Spine (Phila Pa 1976). 09/01 2008;33(19):2108-2115.

477. Tosteson ANA, Tosteson TD, Lurie JD, et al. Comparative effectiveness evidence from the spine patient outcomes research trial: surgical versus nonoperative care for spinal stenosis, degenerative spondylolisthesis, and intervertebral disc herniation. Spine (Phila Pa 1976). 11/15 2011;36(24):2061-2068.

478. Tuominen U, Sintonen H, Hirvonen J, et al. Is longer waiting time for total knee replacement associated with health outcomes and medication costs? Randomized clinical trial. Value Health. 12/ 2010;13(8):998-991004.

479. Tykka E, Rasanen P, Tukiainen E, et al. Cost-utility of breast reduction surgery--a prospective study. J Plast Reconstr Aesthet Surg. 01/ 2010;63(1):87-92.

480. Vaidya A, Borgonovi E, Taylor RS, et al. The cost-effectiveness of the Argus II retinal prosthesis in Retinitis Pigmentosa patients. BMC Ophthalmol. / 2014;14:49-49.

481. Valldeoriola F, Morsi O, Tolosa E, Rumi‡ J, Marti MJ, Martinez-Martin P. Prospective comparative study on cost-effectiveness of subthalamic stimulation and best medical treatment in advanced Parkinson's disease. Mov Disord. 11/15 2007;22(15):2183-2191.

482. van Breugel NH, Bidar E, Essers BA, et al. Cost-effectiveness of ablation surgery in patients with atrial fibrillation undergoing cardiac surgery. Interact Cardiovasc Thorac Surg. 03/ 2011;12(3):394-398.

483. van den Akker ME, Arts MP, van den Hout WB, Brand R, Koes BW, Peul WC. Tubular diskectomy vs conventional microdiskectomy for the treatment of lumbar disk-related sciatica: cost utility analysis alongside a double-blind randomized controlled trial. Neurosurgery. 10/ 2011;69(4):829-835.

484. Van Den Brink M, Van Den Hout WB, Stiggelbout AM, et al. Cost-utility analysis of preoperative radiotherapy in patients with rectal cancer undergoing total mesorectal excision: a study of the Dutch Colorectal Cancer Group. J Clin Oncol. 01/15 2004;22(2):244-253.

485. van den Broek FJC, de Graaf EJR, Dijkgraaf MGW, et al. Transanal endoscopic microsurgery versus endoscopic mucosal resection for large rectal adenomas (TREND-study). BMC Surg. / 2009;9:4-4.

486. van den Hout WB, Peul WC, Koes BW, Brand R, Kievit J, Thomeer RTWM. Prolonged conservative care versus early surgery in patients with sciatica from lumbar disc herniation: cost utility analysis alongside a randomised controlled trial. BMJ. 06/14 2008;336(7657):1351-1354.

487. van der Sluis FJF, Bosch JL, Terkivatan T, de Man RA, Ijzermans JNM, Hunink MGM. Hepatocellular adenoma: cost-effectiveness of different treatment strategies. Radiology. 09/ 2009;252(3):737-746.

488. van Enckevort PJ, Koopmanschap MA, Tenvergert EM, et al. Lifetime costs of lung transplantation: estimation of incremental costs. Health Econ. / 1997;6(5):479-489.

489. van Gemert WG, Adang EM, Kop M, Vos G, Greve JW, Soeters PB. A prospective cost-effectiveness analysis of vertical banded gastroplasty for the treatment of morbid obesity. Obes Surg. 10/ 1999;9(5):484-491.

490. van Mastrigt GAPG, van Dielen FMH, Severens JL, Voss GBWE, Greve JW. One-year cost-effectiveness of surgical treatment of morbid obesity: vertical banded gastroplasty versus Lap-Band. Obes Surg. 01/ 2006;16(1):75-84.

491. van Wunnik BPW, Visschers RGJ, van Asselt ADI, Baeten CGMI. Cost-effectiveness analysis of sacral neuromodulation for faecal incontinence in The Netherlands. Colorectal Dis. 12/ 2012;14(12):807-814.

492. Vasiliadis H-M, Collet J-P, Penrod JR, Ferraro P, Poirier C. A cost-effectiveness and cost-utility study of lung transplantation. J Heart Lung Transplant. 09/ 2005;24(9):1275-1283.

493. Verguet S, Stalcup M, Walsh JA. Where to deploy pre-exposure prophylaxis (PrEP) in sub-Saharan Africa? Sex Transm Infect. 12/ 2013;89(8):628-634.

494. Vidal-Trecan GM, Stahl JE, Eckman MH. Radioiodine or surgery for toxic thyroid adenoma: dissecting an important decision. A cost-effectiveness analysis. Thyroid. 11/ 2004;14(11):933-945.

495. Vieira RDO, Hueb W, Hlatky M, et al. Cost-effectiveness analysis for surgical, angioplasty, or medical therapeutics for coronary artery disease: 5-year follow-up of medicine, angioplasty, or surgery study (MASS) II trial. Circulation. 09/11 2012;126(11 Suppl 1):145-150.

496. Vij R, Triadafilopoulos G, Owens DK, Kunz P, Sanders GD. Cost-effectiveness of photodynamic therapy for high-grade dysplasia in Barrett's esophagus. Gastrointest Endosc. 11/ 2004;60(5):739-756.

497. Vilain KR, Magnuson EA, Li H, et al. Costs and cost-effectiveness of carotid stenting versus endarterectomy for patients at standard surgical risk: results from the Carotid Revascularization Endarterectomy Versus Stenting Trial (CREST). Stroke. 09/ 2012;43(9):2408-2416.

498. Visser K, de Vries SO, Kitslaar PJEHM, van Engelshoven JMA, Hunink MGM. Cost-effectiveness of diagnostic imaging work-up and treatment for patients with intermittent claudication in The Netherlands. Eur J Vasc Endovasc Surg. 03/ 2003;25(3):213-223.

499. Waaler Bjornelv GM, Frihagen F, Madsen JE, Nordsletten L, Aas E. Hemiarthroplasty compared to internal fixation with percutaneous cannulated screws as treatment of displaced femoral neck fractures in the elderly: cost-utility analysis performed alongside a randomized, controlled trial. Osteoporos Int. 06/ 2012;23(6):1711-1719.

500. Wagner TH, Hattler B, Bishawi M, et al. On-pump versus off-pump coronary artery bypass surgery: cost-effectiveness analysis alongside a multisite trial. Ann Thorac Surg. 09/ 2013;96(3):770-777.

501. Wang BCM, Wong ES, Alfonso-Cristancho R, et al. Cost-effectiveness of bariatric surgical procedures for the treatment of severe obesity. Eur J Health Econ. 04/ 2014;15(3):253-263.

502. Wang L, Wen H, Feng X, Jiang X, Duan X. Analysis of economic burden for patients with cystic echinococcosis in five hospitals in northwest China. Trans R Soc Trop Med Hyg. 12/ 2012;106(12):743-748.

503. Warf BC, Alkire BC, Bhai S, et al. Costs and benefits of neurosurgical intervention for infant hydrocephalus in sub-Saharan Africa. J Neurosurg Pediatr. 11/ 2011;8(5):509-521.

504. Warren D, Andres T, Hoelscher C, Ricart-Hoffiz P, Bendo J, Goldstein J. Cost-utility analysis modeling at 2-year follow-up for cervical disc arthroplasty versus anterior cervical discectomy and fusion: A single-center contribution to the randomized controlled trial. International Journal of Spine Surgery. 2013;7(1):e58-e66.

505. Warren D, Hoelscher C, Ricart-Hoffiz P, Bendo J, Goldstein J. Cost-utility analysis of anterior cervical discectomy and fusion versus cervical disc arthroplasty. Evid Based Spine Care J. 08/ 2011;2(3):57-58.

506. Watt M, Mealing S, Eaton J, et al. Cost-effectiveness of transcatheter aortic valve replacement in patients ineligible for conventional aortic valve replacement. Heart. 03/ 2012;98(5):370-376.

507. Watters TS, Browne JA, Orlando LA, Wellman SS, Urbaniak JR, Bolognesi MP. Cost-effectiveness analysis of free vascularized fibular grafting for osteonecrosis of the femoral head. J Surg Orthop Adv. / 2011;20(3):158-167.

508. Weinstock B, Dattilo R, Diage T. Cost-effectiveness analysis of orbital atherectomy plus balloon angioplasty vs balloon angioplasty alone in subjects with calcified femoropopliteal lesions. Clinicoecon Outcomes Res. / 2014;6:133-139.

509. Weintraub WS, Boden WE, Zhang Z, et al. Cost-effectiveness of percutaneous coronary intervention in optimally treated stable coronary patients. Circ Cardiovasc Qual Outcomes. 09/ 2008;1(1):12-20.

510. Welcker K, Lederle J, Schorr M, Siebeck M. Surgery and adjuvant therapy in patients with diffuse peritonitis: cost analysis. World J Surg. 03/ 2002;26(3):307-313.

511. Welcker K, Marian P, Thetter O, Siebeck M. Cost and quality of life in thoracic surgery--a health economic analysis in a German center. Thorac Cardiovasc Surg. 10/ 2003;51(5):260-266.

512. Whiting JF, Kiberd B, Kalo Z, Keown P, Roels L, Kjerulf M. Cost-effectiveness of organ donation: evaluating investment into donor action and other donor initiatives. Am J Transplant. 04/ 2004;4(4):569-573.

513. Whitmore RG, Thawani JP, Grady MS, Levine JM, Sanborn MR, Stein SC. Is aggressive treatment of traumatic brain injury cost-effective? J Neurosurg. 05/ 2012;116(5):1106-1113.

514. Wijeysundera HC, Tomlinson G, Ko DT, Dzavik V, Krahn MD. Medical therapy v. PCI in stable coronary artery disease: a cost-effectiveness analysis. Med Decis Making. 10/ 2013;33(7):891-905.

515. Will BP, Berthelot JM, Nobrega KM, Flanagan W, Evans WK. Canada's Population Health Model (POHEM): a tool for performing economic evaluations of cancer control interventions. Eur J Cancer. 09/ 2001;37(14):1797-1804.

516. Williams A. Economics of coronary artery bypass grafting. Br Med J (Clin Res Ed). 08/03 1985;291(6491):326-329.

517. Wong CKH, Lang BH-H. A cost-utility analysis for prophylactic central neck dissection in clinically nodal-negative papillary thyroid carcinoma. Ann Surg Oncol. 03/ 2014;21(3):767-777.

518. Wu VK, Poenaru D. Burden of surgically correctable disabilities among children in the Dadaab Refugee Camp. World J Surg. 07/ 2013;37(7):1536-1543.

519. Wu Y, Jin R, Gao G, Grunkemeier GL, Starr A. Cost-effectiveness of aortic valve replacement in the elderly: an introductory study. J Thorac Cardiovasc Surg. 03/ 2007;133(3):608-613.

520. Wyatt JR, Niparko JK, Rothman M, deLissovoy G. Cost utility of the multichannel cochlear implants in 258 profoundly deaf individuals. Laryngoscope. 07/ 1996;106(7):816-821.

521. Wymer KM, Shih Y-CT, Plunkett BA. The cost-effectiveness of a trial of labor accrues with multiple subsequent vaginal deliveries. Am J Obstet Gynecol. 07/ 2014;211(1):1-56.

522. Xie F, Lo N-N, Tarride J-E, O'Reilly D, Goeree R, Lee H-P. Total or partial knee replacement? Cost-utility analysis in patients with knee osteoarthritis based on a 2-year observational study. Eur J Health Econ. 02/ 2010;11(1):27-34.

523. Xu X, Ivy JS, Patel DA, et al. Pelvic floor consequences of cesarean delivery on maternal request in women with a single birth: a cost-effectiveness analysis. J Womens Health (Larchmt). 01/ 2010;19(1):147-160.

524. Xue H, Lacson E, Wang W, Curhan GC, Brunelli SM. Choice of vascular access among incident hemodialysis patients: a decision and cost-utility analysis. Clin J Am Soc Nephrol. 12/ 2010;5(12):2289-2296.

525. Yaghoubi M, Aghayan HR, Arjmand B, Emami-Razavi SH. Cost-effectiveness of homograft heart valve replacement surgery: an introductory study. Cell Tissue Bank. 05/ 2011;12(2):153-158.

526. Yeh JM, Ho W, Hur C. Cost-effectiveness of endoscopic surveillance of gastric ulcers to improve survival. Gastrointest Endosc. 07/ 2010;72(1):33-43.

527. Yianni J, Green AL, McIntosh E, et al. The costs and benefits of deep brain stimulation surgery for patients with dystonia: an initial exploration. Neuromodulation. 07/ 2005;8(3):155-161.

528. Yiee JH, Baskin LS. Use of internal stent, external transanastomotic stent or no stent during pediatric pyeloplasty: a decision tree cost-effectiveness analysis. J Urol. 02/ 2011;185(2):673-680.

529. Yin D, Carpenter JP. Cost-effectiveness of screening for asymptomatic carotid stenosis. J Vasc Surg. 02/ 1998;27(2):245-255.

530. Yock CA, Boothroyd DB, Owens DK, Garber AM, Hlatky MA. Cost-effectiveness of bypass surgery versus stenting in patients with multivessel coronary artery disease. Am J Med. 10/01 2003;115(5):382-389.

531. You JHS, Sahota DS, MoYuen P. A cost-utility analysis of hysterectomy, endometrial resection and ablation and medical therapy for menorrhagia. Hum Reprod. 07/ 2006;21(7):1878-1883.

532. You JHS, Sahota DS, Yuen PM. Uterine artery embolization, hysterectomy, or myomectomy for symptomatic uterine fibroids: a cost-utility analysis. Fertil Steril. 02/ 2009;91(2):580-588.

533. Young KC, Awad NA, Johansson M, Gillespie D, Singh MJ, Illig KA. Cost-effectiveness of abdominal aortic aneurysm repair based on aneurysm size. J Vasc Surg. 01/ 2010;51(1):27-32.

534. Young KC, Holloway RG, Burgin WS, Benesch CG. A cost-effectiveness analysis of carotid artery stenting compared with endarterectomy. J Stroke Cerebrovasc Dis. / 2010;19(5):404-409.

535. Young KC, Teeters JC, Benesch CG, Bisognano JD, Illig KA. Cost-effectiveness of treating resistant hypertension with an implantable carotid body stimulator. J Clin Hypertens (Greenwich). 10/ 2009;11(10):555-563.

536. Zanocco K, Elaraj D, Sturgeon C. Routine prophylactic central neck dissection for low-risk papillary thyroid cancer: a cost-effectiveness analysis. Surgery. 12/ 2013;154(6):1148-1155.

537. Zanocco K, Heller M, Elaraj D, Sturgeon C. Cost effectiveness of intraoperative pathology examination during diagnostic hemithyroidectomy for unilateral follicular thyroid neoplasms. J Am Coll Surg. 10/ 2013;217(4):702-710.

538. Zelle SG, Nyarko KM, Bosu WK, et al. Costs, effects and cost-effectiveness of breast cancer control in Ghana. Trop Med Int Health. 08/ 2012;17(8):1031-1043.

539. Zendejas B, Moriarty JP, O'Byrne J, Degnim AC, Farley DR, Boughey JC. Cost-effectiveness of contralateral prophylactic mastectomy versus routine surveillance in patients with unilateral breast cancer. J Clin Oncol. 08/01 2011;29(22):2993-3000.

540. Zowall H, Cairns JA, Brewer C, Lamping DL, Gedroyc WMW, Regan L. Cost-effectiveness of magnetic resonance-guided focused ultrasound surgery for treatment of uterine fibroids. BJOG. 04/ 2008;115(5):653-662.
